# Supplementary material for: Mapping signal transduction in bistable jumping spider rhodopsin 1
Source: Biophys J. 2025 Nov 3;125(10):2287–97. doi: 10.1016/j.bpj.2025.10.040 (PMC13351664; doi:10.1016/j.bpj.2025.10.040)
Supplement: Document S1. Figures S1–S17 and Tables S1–S5 [file mmc1.pdf]

Biophysical Journal, Volume *125*

Supplemental information

Mapping signal transduction in bistable jumping spider rhodopsin 1

Flavio Costa, Emanuele Telari, Daniel Moreno-Rodríguez, Simone Meloni, Jógvan Magnus Haugaard Olsen, Alberto Giacomello, and Giovanni Di Muccio

Supporting Information: Mapping Signal Transduction in Bistable Jumping Spider Rhodopsin 1

Flavio Costa,[†] Emanuele Telari,[‡] Daniel Moreno-Rodríguez,[†] Simone Meloni,[¶] Jógvan Magnus Haugaard Olsen,[§] Alberto Giacomello,[†] and Giovanni Di Muccio*,[†,‖]

†*Dipartimento di Ingegneria Meccanica e Aerospaziale, Sapienza Università di Roma, Via Eudossiana 18, 00184, Rome, Italy*

‡*Departament de Ciència de Materials i Química Física & Institut de Quimica Teòrica i Computacional (IQTCUB), Universitat de Barcelona, c/Martí i Franquès 1, 08028 Barcelona, Spain*

¶*Department of Chemical, Pharmaceutical and Agricultural Sciences, University of Ferrara, Ferrara, 44121 Italy*

§*DTU Chemistry, Technical University of Denmark, DK-2800 Kongens Lyngby, Denmark*

‖*NY-Masbic, Department of Life and Environmental Sciences, Marche Polytechnic University, Via Brecce Bianche, 60131, Ancona, Italy*

E-mail: g.dimuccio@univpm.it

```
----------------------------------------------------------------
        Layer (type)               Output Shape         Param #
================================================================
            Conv2d-1         [-1, 16, 130, 130]             160
         MaxPool2d-2           [-1, 16, 65, 65]               0
              ReLU-3           [-1, 16, 65, 65]               0
       BatchNorm2d-4           [-1, 16, 65, 65]              32
            Conv2d-5           [-1, 32, 65, 65]           4,640
         MaxPool2d-6           [-1, 32, 32, 32]               0
              ReLU-7           [-1, 32, 32, 32]               0
       BatchNorm2d-8           [-1, 32, 32, 32]              64
            Conv2d-9           [-1, 64, 32, 32]          18,496
        MaxPool2d-10           [-1, 64, 16, 16]               0
             ReLU-11           [-1, 64, 16, 16]               0
      BatchNorm2d-12           [-1, 64, 16, 16]             128
           Conv2d-13           [-1, 64, 16, 16]          36,928
        MaxPool2d-14             [-1, 64, 8, 8]               0
             ReLU-15             [-1, 64, 8, 8]               0
      BatchNorm2d-16             [-1, 64, 8, 8]             128
           Conv2d-17             [-1, 64, 8, 8]          36,928
        MaxPool2d-18             [-1, 64, 4, 4]               0
             ReLU-19             [-1, 64, 4, 4]               0
      BatchNorm2d-20             [-1, 64, 4, 4]             128
           Conv2d-21            [-1, 128, 4, 4]          73,856
        MaxPool2d-22            [-1, 128, 2, 2]               0
             ReLU-23            [-1, 128, 2, 2]               0
      BatchNorm2d-24            [-1, 128, 2, 2]             256
          Flatten-25                  [-1, 512]               0
           Linear-26                    [-1, 2]           1,026
           Linear-27                  [-1, 512]           1,536
             ReLU-28                  [-1, 512]               0
         Upsample-29            [-1, 128, 4, 4]               0
  ConvTranspose2d-30             [-1, 64, 4, 4]          73,792
             ReLU-31             [-1, 64, 4, 4]               0
      BatchNorm2d-32             [-1, 64, 4, 4]             128
         Upsample-33             [-1, 64, 8, 8]               0
  ConvTranspose2d-34             [-1, 64, 8, 8]          36,928
             ReLU-35             [-1, 64, 8, 8]               0
      BatchNorm2d-36             [-1, 64, 8, 8]             128
         Upsample-37           [-1, 64, 16, 16]               0
  ConvTranspose2d-38           [-1, 64, 16, 16]          36,928
             ReLU-39           [-1, 64, 16, 16]               0
      BatchNorm2d-40           [-1, 64, 16, 16]             128
         Upsample-41           [-1, 64, 32, 32]               0
  ConvTranspose2d-42           [-1, 32, 32, 32]          18,464
             ReLU-43           [-1, 32, 32, 32]               0
      BatchNorm2d-44           [-1, 32, 32, 32]              64
         Upsample-45           [-1, 32, 64, 64]               0
  ConvTranspose2d-46           [-1, 16, 64, 64]           4,624
             ReLU-47           [-1, 16, 64, 64]               0
      BatchNorm2d-48           [-1, 16, 64, 64]              32
         Upsample-49         [-1, 16, 128, 128]               0
           Conv2d-50          [-1, 1, 130, 130]             145
================================================================
Total params: 345,667
Trainable params: 345,667
Non-trainable params: 0
----------------------------------------------------------------
Input size (MB): 0.06
Forward/backward pass size (MB): 13.12
Params size (MB): 1.32
Estimated Total Size (MB): 14.51
----------------------------------------------------------------
```

Figure S1: Network configuration as printed by torchsummary. The matrices fed to the network were padded with a row and column of zeros to reach a size of 130×130, from the initial size of 129×129, being this one more complicated to handle with the max poolings and upscalings

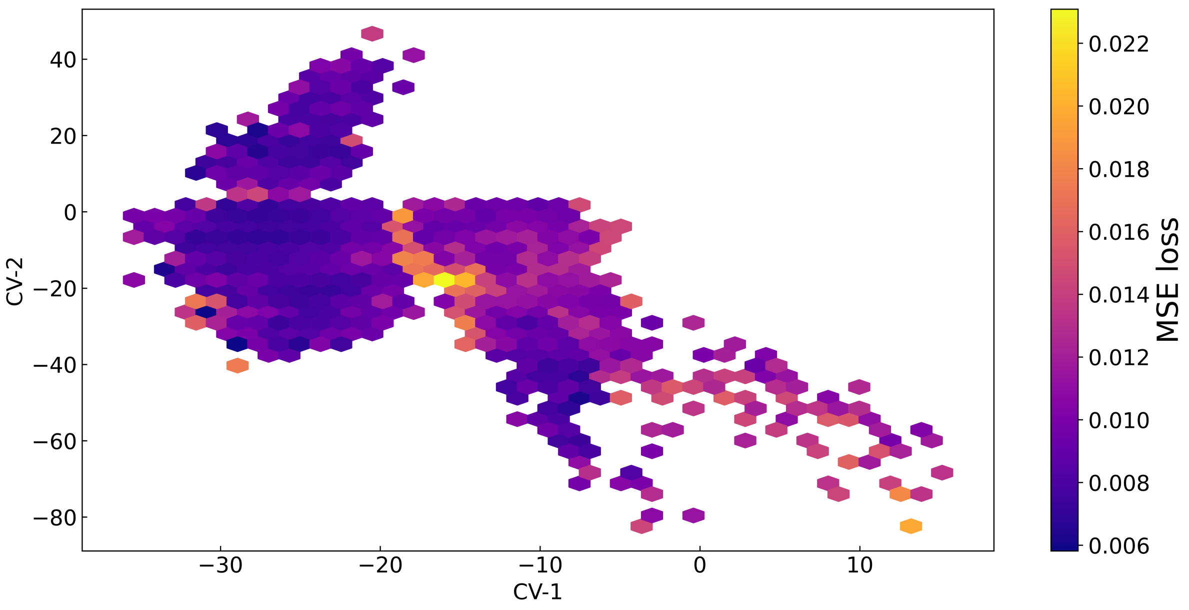

Figure S2: Heat map of the MSE loss values across the reduced space.

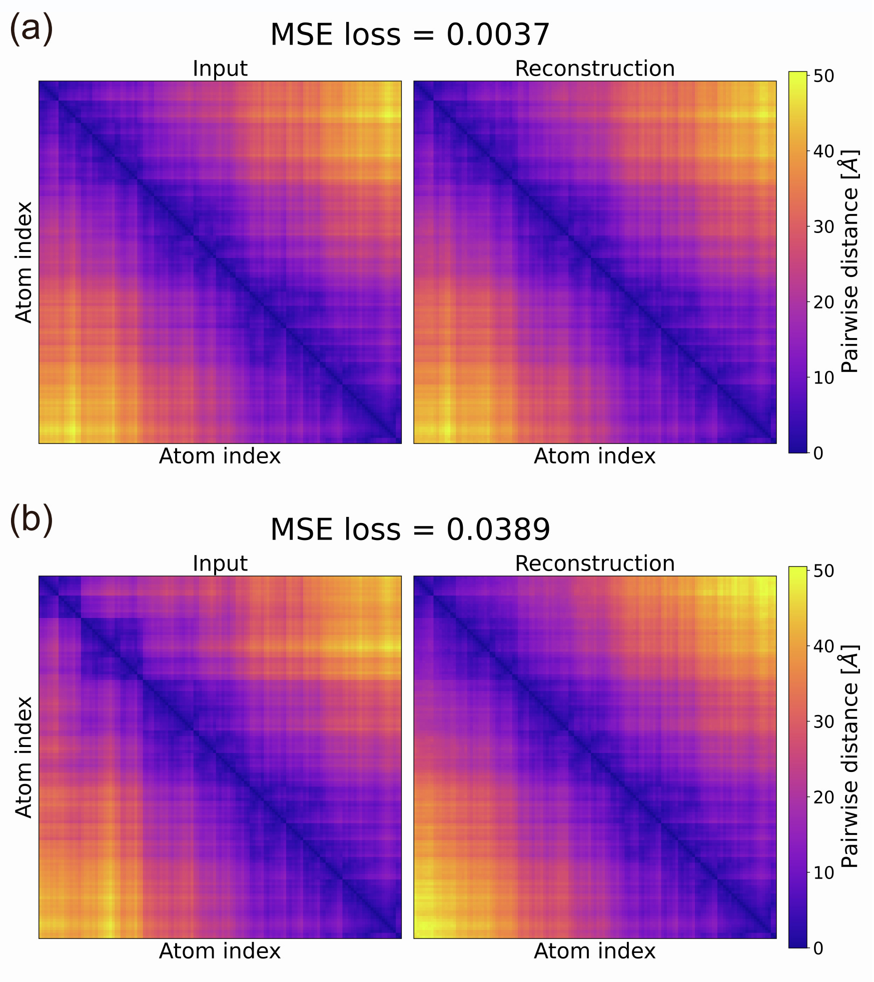

Figure S3: (a) Comparison between input (left) and reconstructed output (right) of the network for the sample associated to the best (lowest) MSE. The two heat maps share the same color scale reported by the colorbar on the right. (b) Comparison between input and output for the sample showing the highest MSE value. The two heat maps share the same color scale reported by the colorbar on the right.

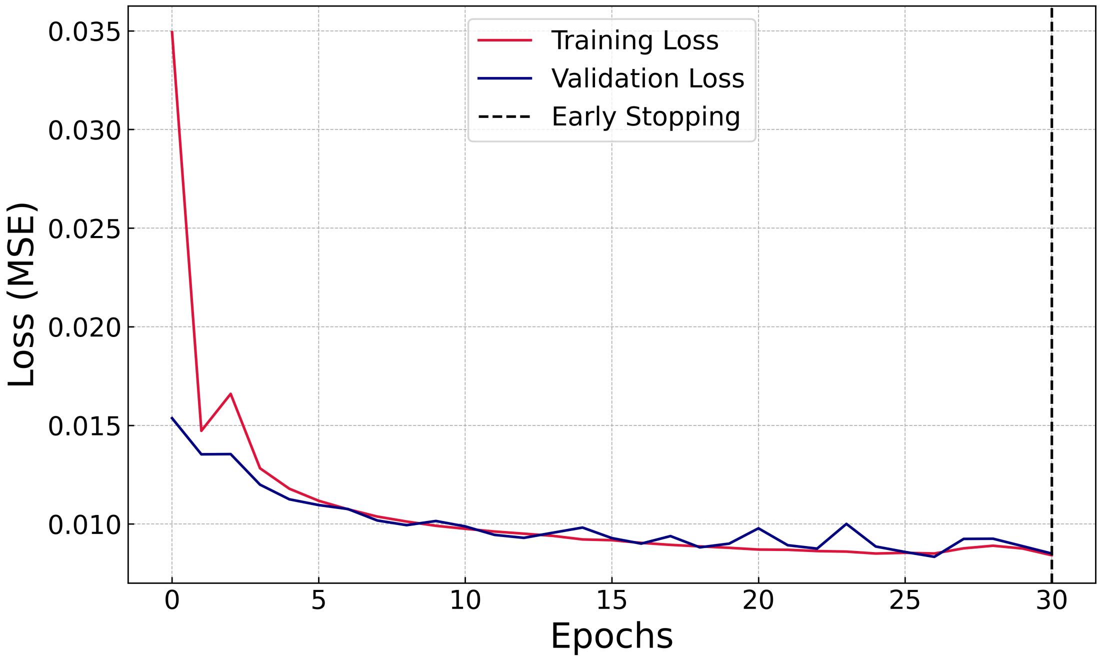

Figure S4: Plots of the training and validation loss during the training. The early stopping stopped the training after 30 epochs and the best model saved was the one of epoch 30.

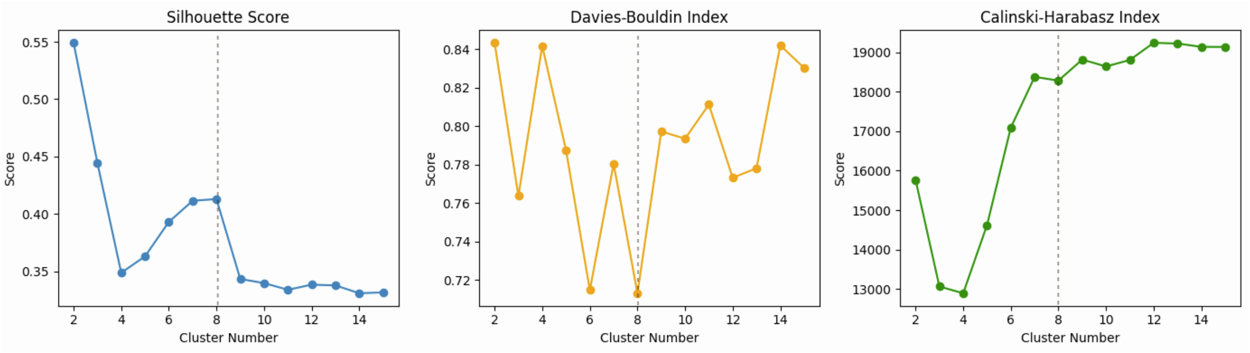

Figure S5: Plot of three metrics used to evaluate the clustering performance and the optimal number of clusters. Dotted lines indicate the final number of clusters used.

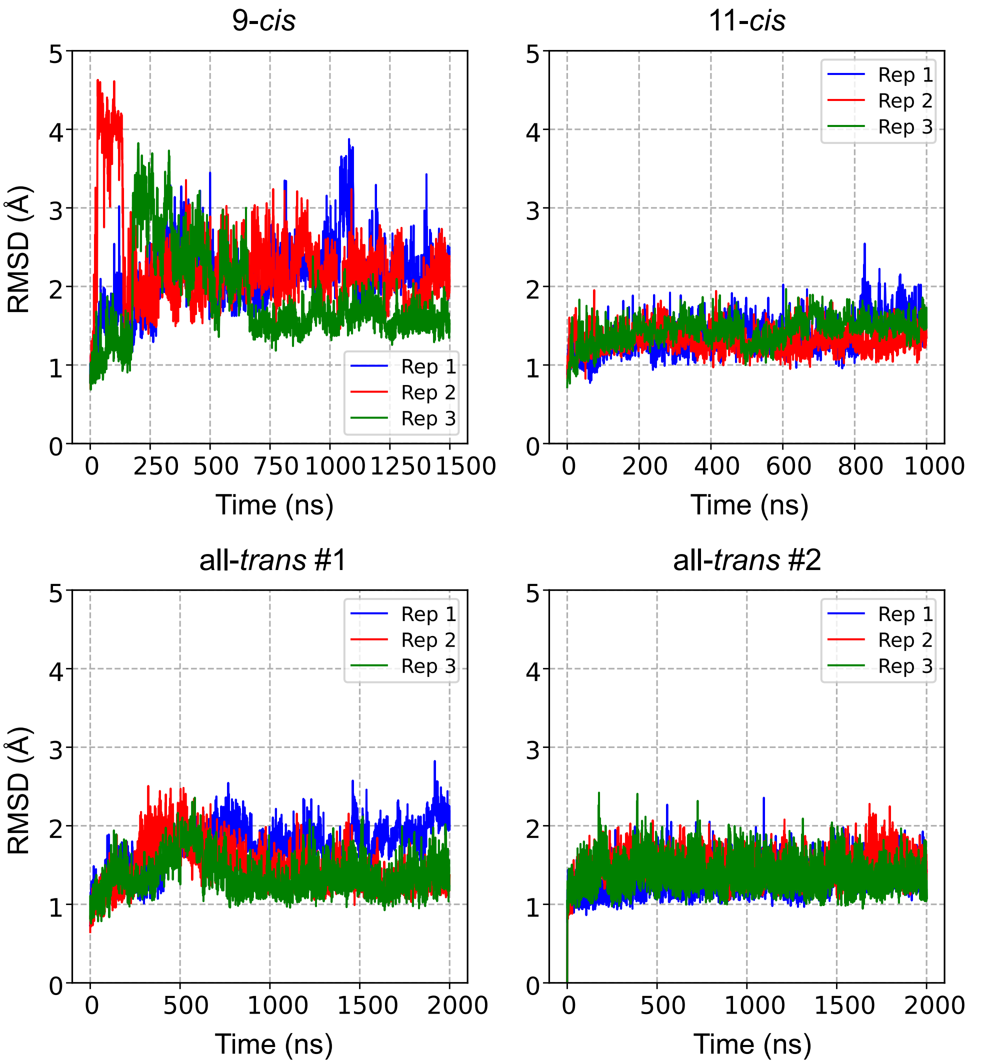

Figure S6: Root Mean Square Displacement calculations of each replica where only the Cα were considered and the reference conformation corresponded to that at the beginning of the dynamics.

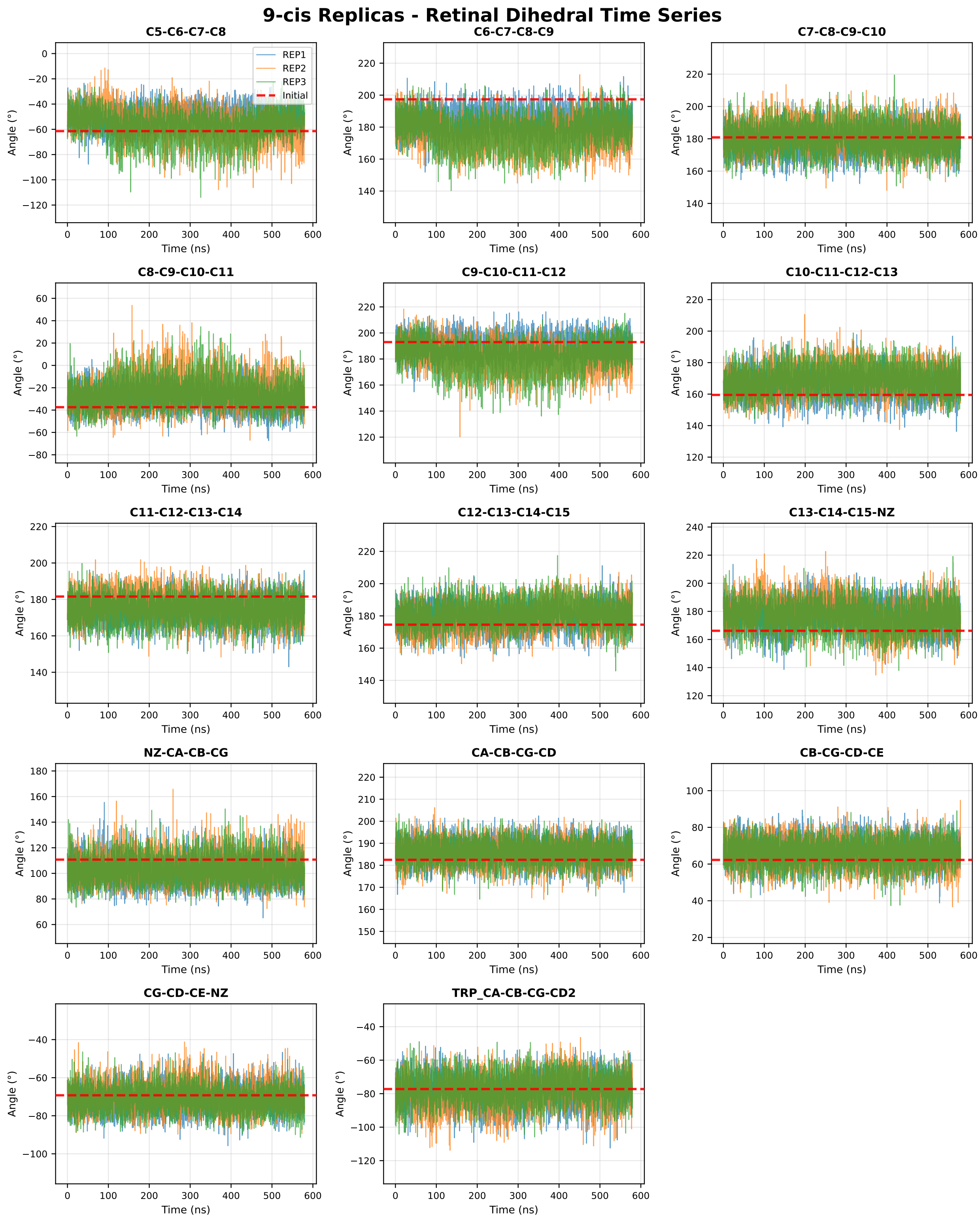

Figure S7: MD retinal dihedral angles for **9-cis** isomer conformation. Starting structure angle is reported in dashed red line. Summary Table is reported in Table S1.

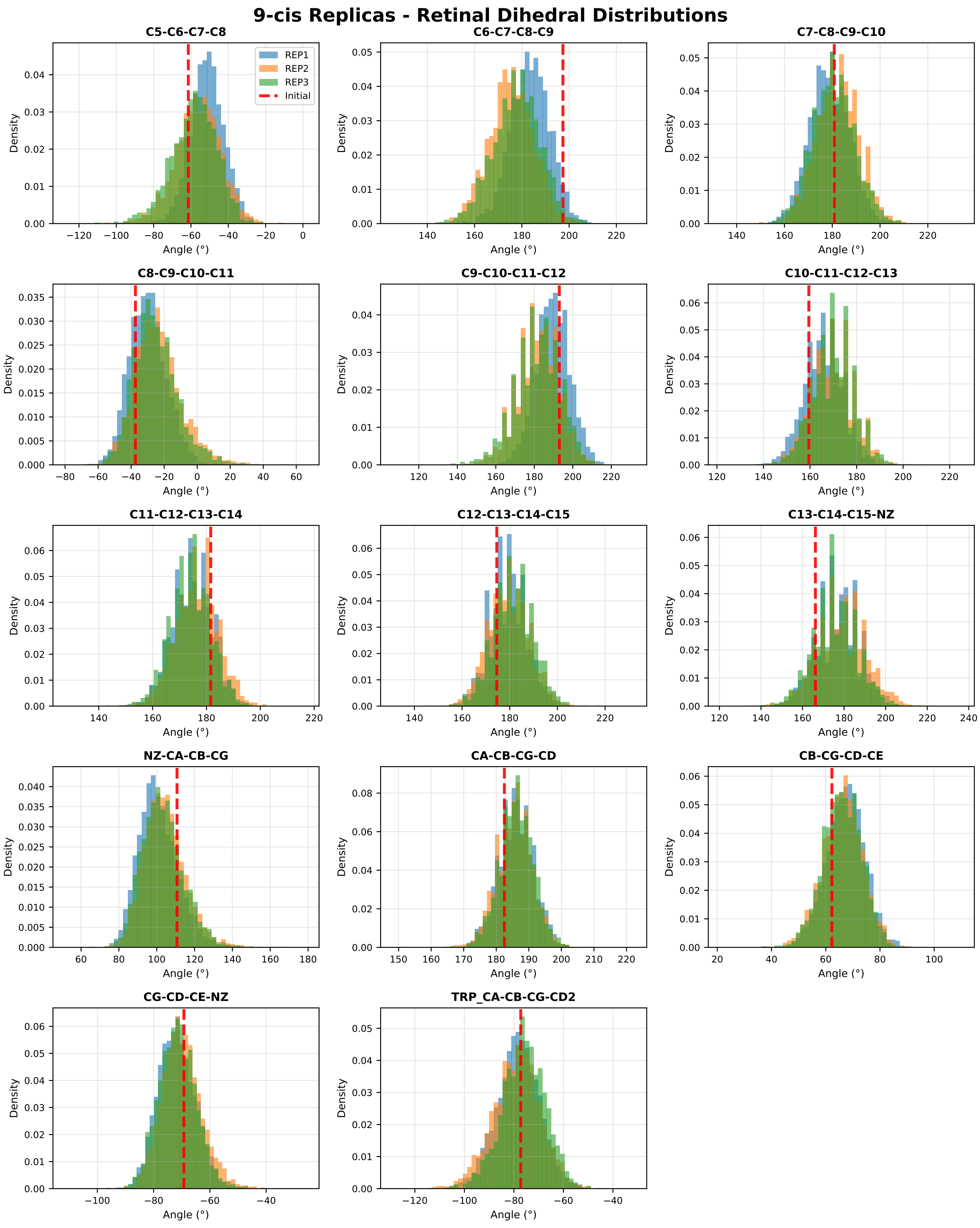

Figure S8: MD retinal dihedral angles for **9-cis** isomer conformation. Starting structure angle is reported in dashed red line. Summary Table is reported in Table S1.

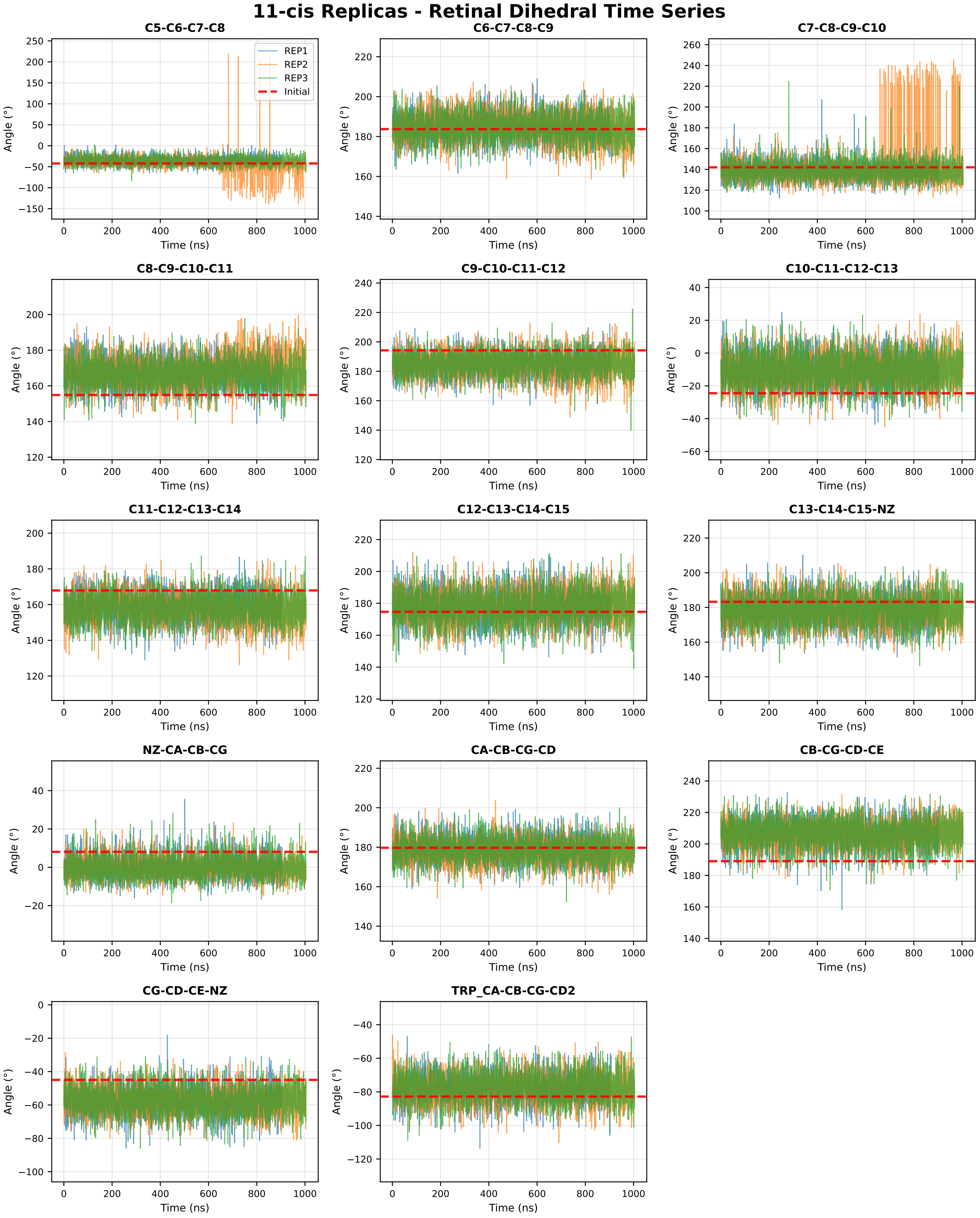

Figure S9: MD retinal dihedral angles for **11-cis** isomer conformation. Starting structure angle is reported in dashed red line. Summary Table is reported in Table S2.

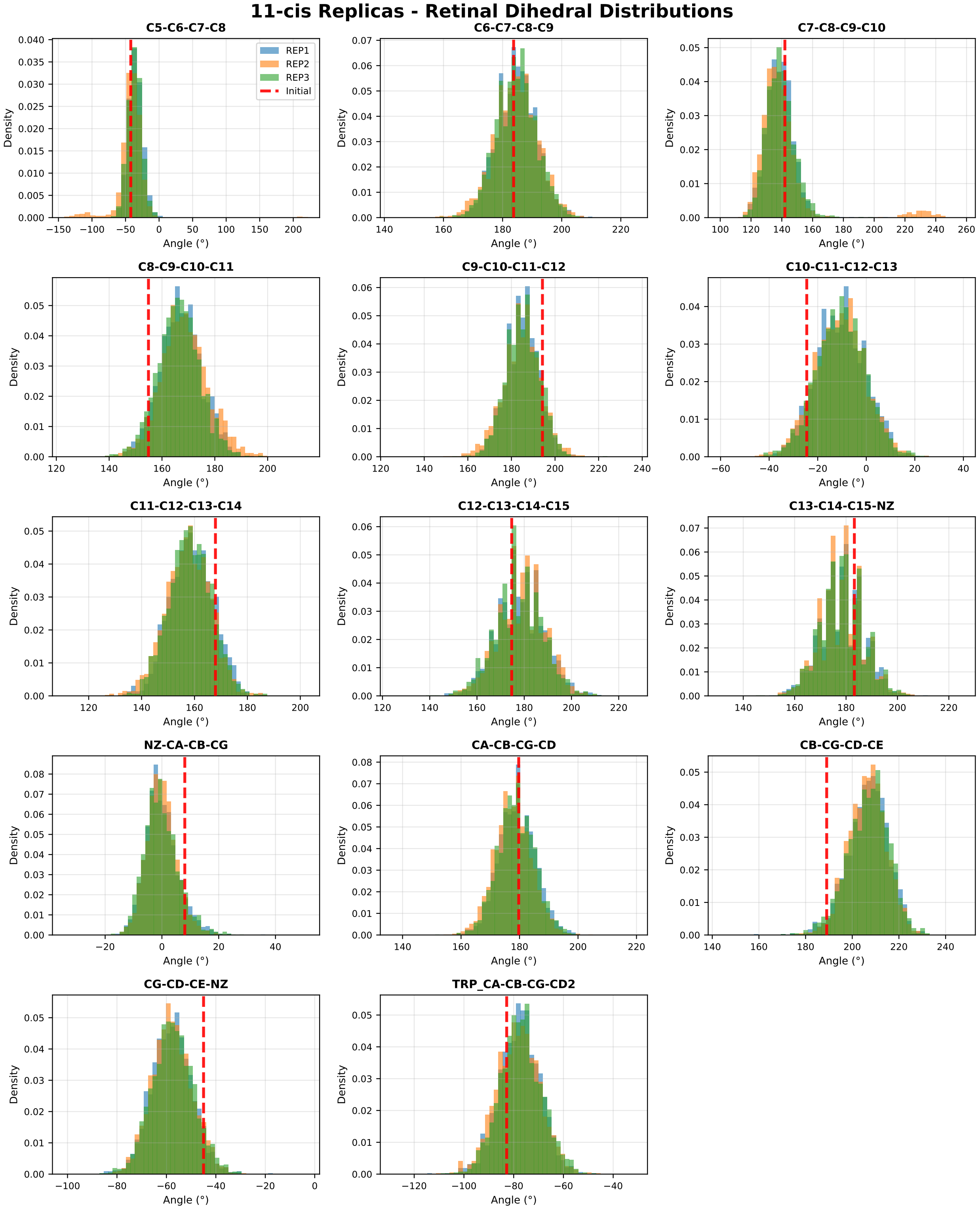

Figure S10: MD retinal dihedral angles for **11-cis** isomer conformation. Starting structure angle is reported in dashed red line. Summary Table is reported in Table S2.

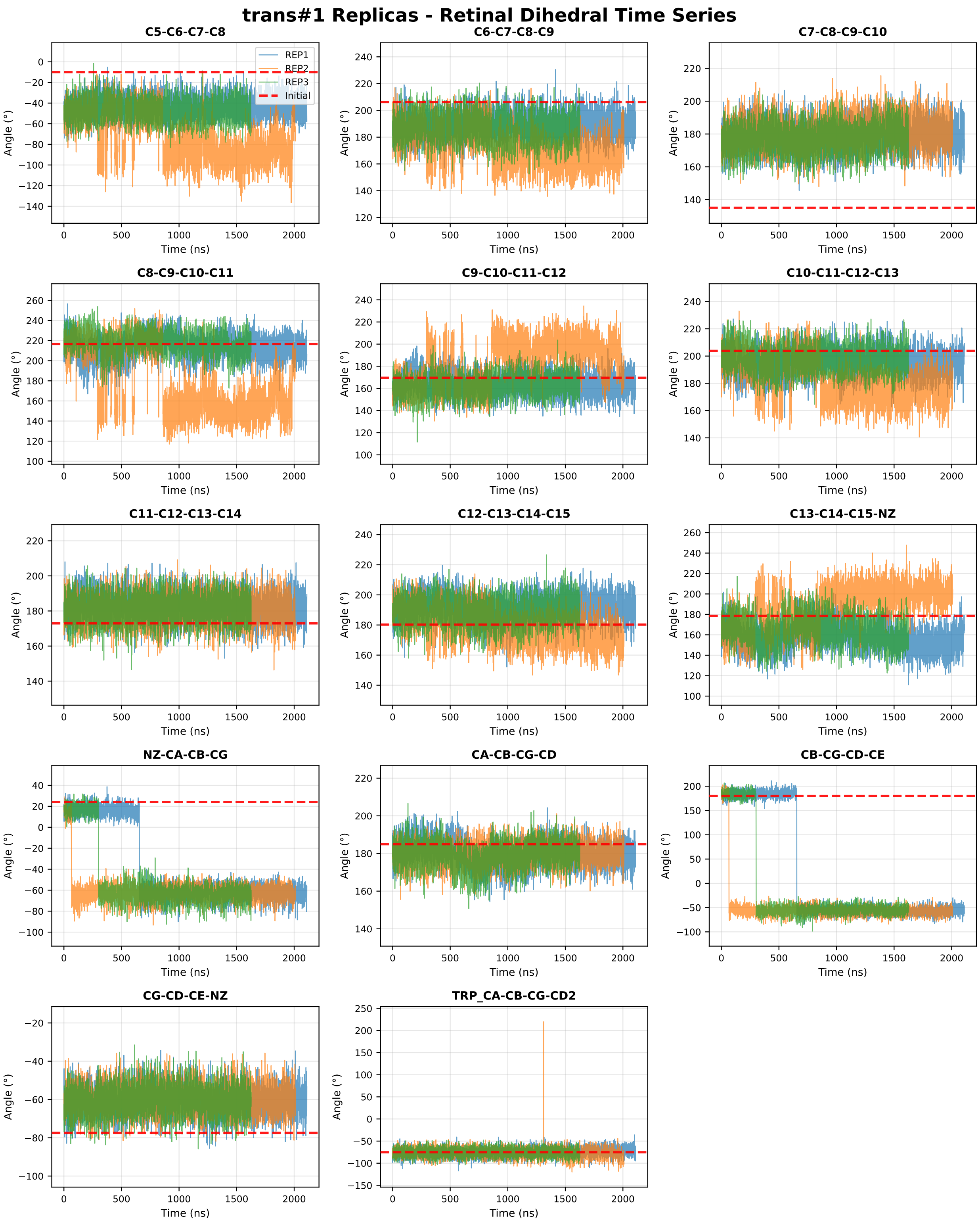

Figure S11: MD retinal dihedral angles for **all-*trans*#1** isomer conformation. Starting structure angle is reported in dashed red line. Summary Table is reported in Table S3.

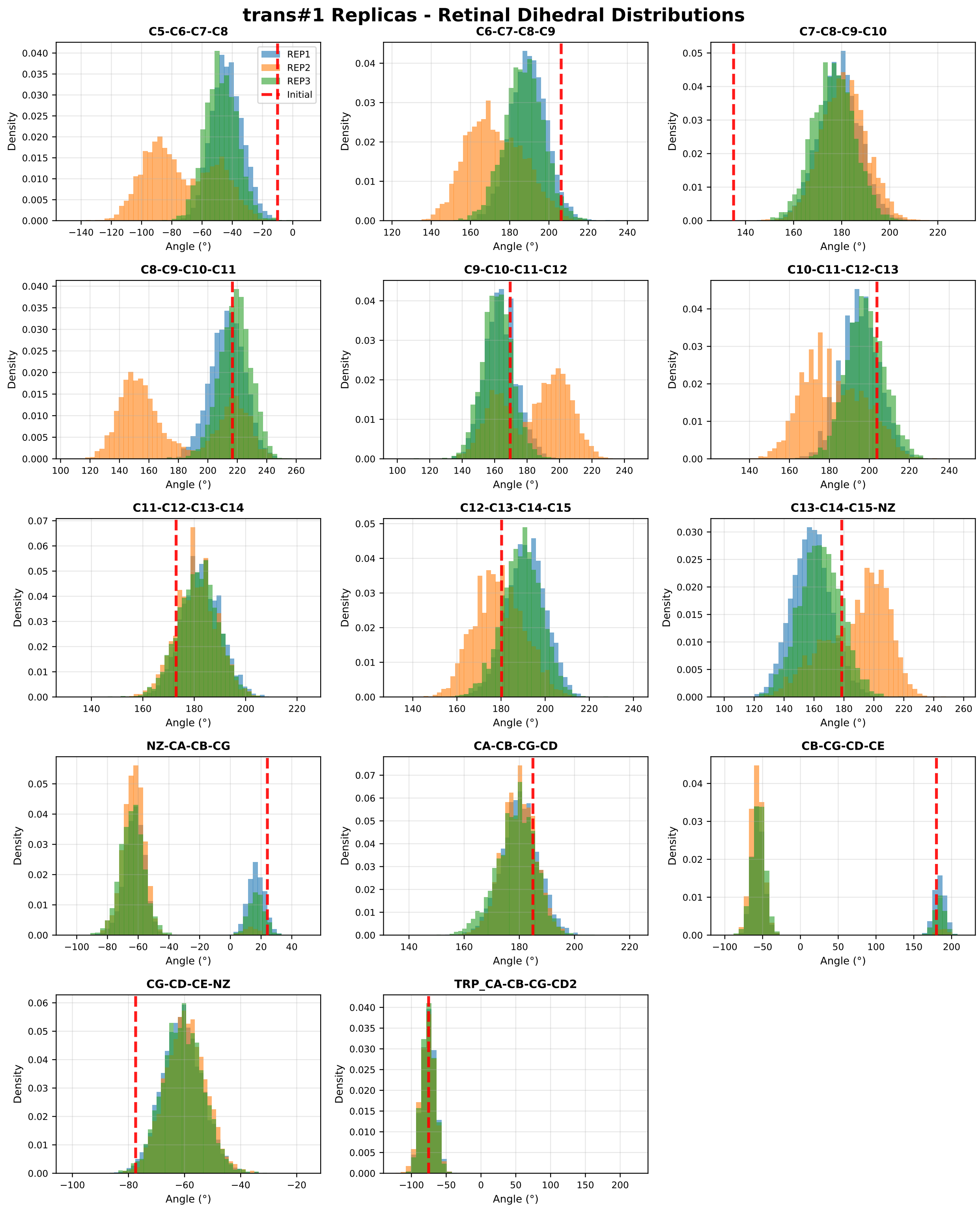

Figure S12: MD retinal dihedral angles for **all-*trans*#1** isomer conformation. Starting structure angle is reported in dashed red line. Summary Table is reported in Table S3.

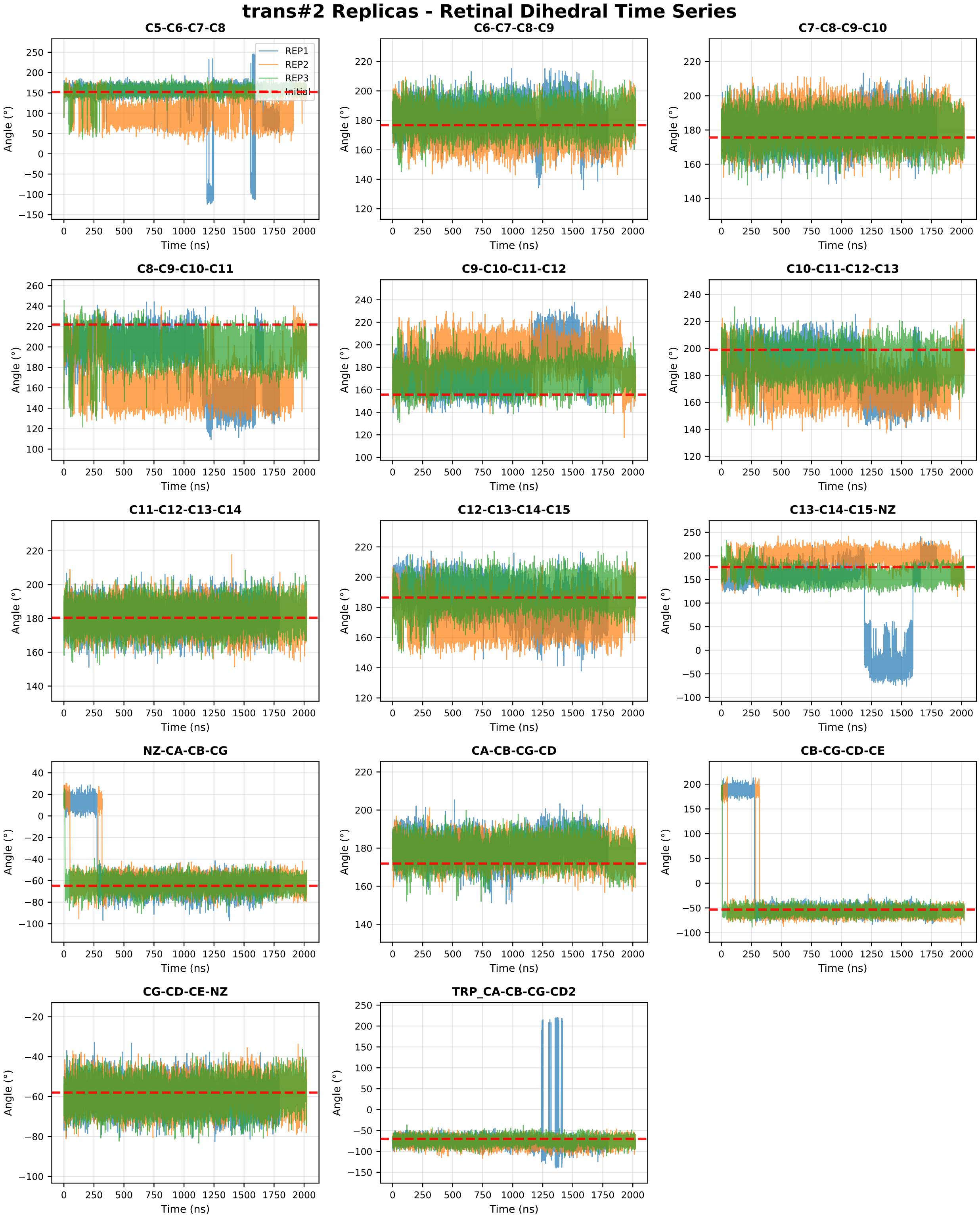

Figure S13: MD retinal dihedral angles for an alternative **all-*trans*#2** isomer conformation. Starting structure angle is reported in dashed red line. Summary Table is reported in Table S4.

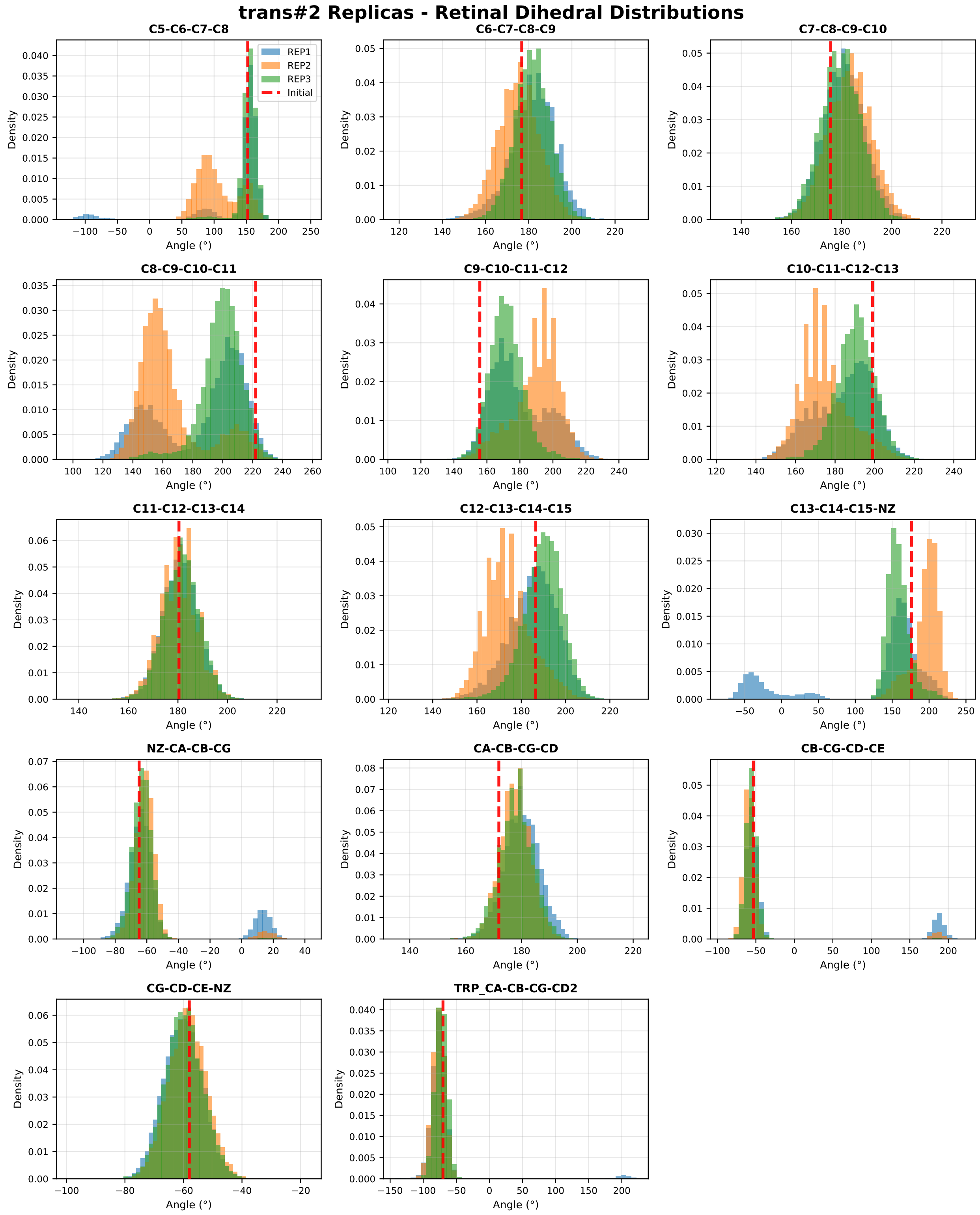

Figure S14: MD retinal dihedral angles for an alternative **all-*trans*#2** isomer conformation. Starting structure angle is reported in dashed red line. Summary Table is reported in Table S4.

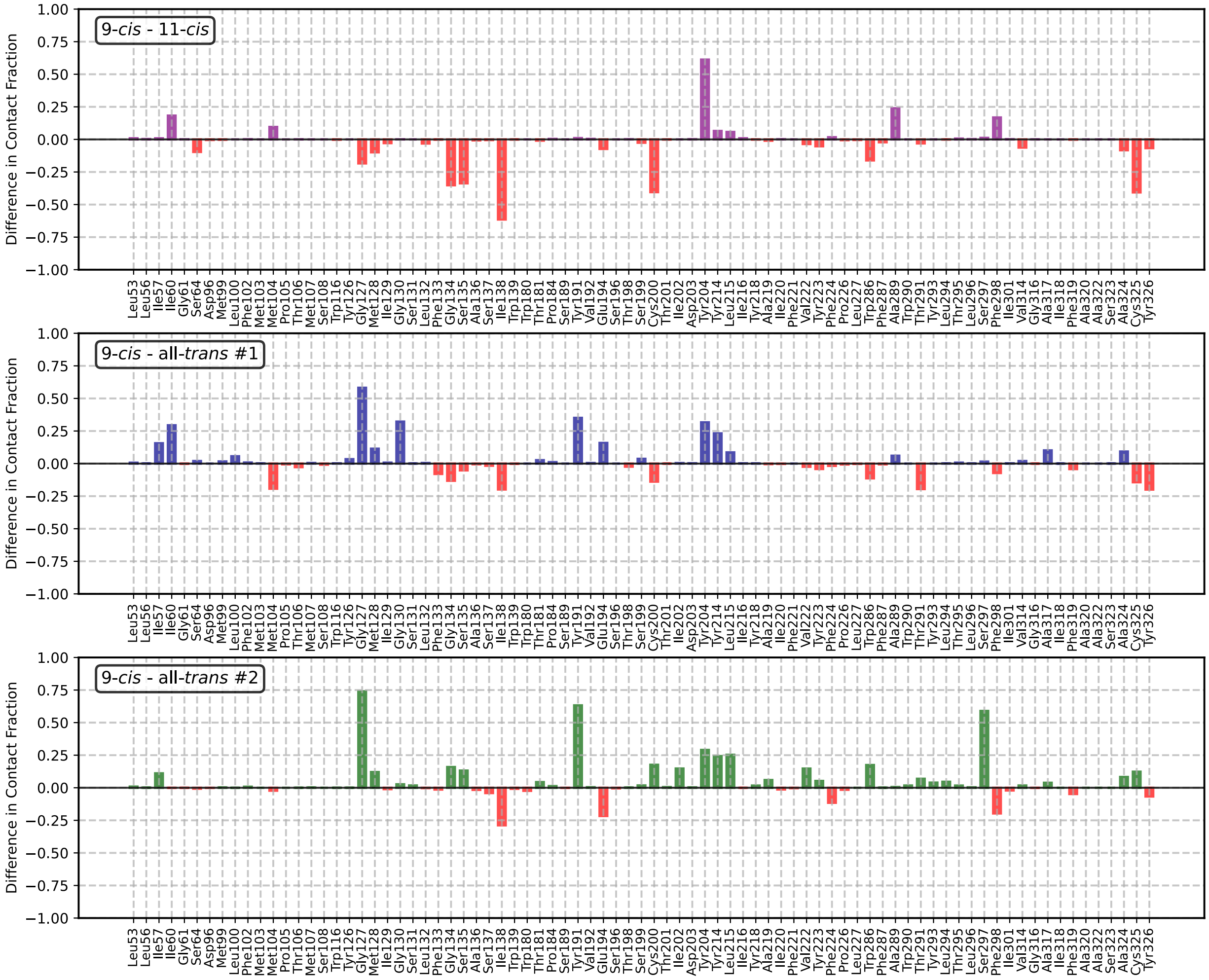

Figure S15: Differences in contact fraction between 9-*cis* and 11-*cis*, all-*trans* #1, and all-*trans* #2 systems. A difference in contact fraction equal to 1 indicates that the interaction persists during the whole trajectory in the 9-*cis* while equal to -1 in the others.

```
Bovine   55 NFLTLYVTVQHKKLRTPLNYILLNLAVADLFMVFGGFTTTLYTSLHGYFVFGPTGCNLEG
JSR1     68 NGVVMYLMMTVKNLRTPGNFLVLNLALSDFGMLFFMMPTMSINCFAETWVIGPFMCELYG
            *    *     * **** *   ****  *  * *    *          * **  * * *

Bovine  115 FFATLGGEIALWSLVVLAIERYVVVCKPMSNFRFGENHAIMGVAFTWVMALACAAPPLVG
JSR1    128 MIGSLFGSASIWSLVMITLDRYNVIVKGMAGKPLTKVGALLRMLFVWIWSLGWTIAPMYG
                * *    ****     ** *  * *         *     * *   *     *  *

Bovine  175 WSRYIPEGMQCSCGIDYYTPHEETNNESFVIYMFVVHFIIPLIVIFFCYGQLVFTVKEAA
JSR1    188 WSRYVPEGSMTSCTIDYIDT--AINPMSYLIAYAIFVYFVPLFIIIYCYAFIVMQVAAHE
            **** ***   ** ***       *  *  *         **  *  **   *  *

Bovine  235 AQQQESATTQK--------------AEKEVTRMVIIMVIAFLICWLPYAGVAFYIFTHQG
JSR1    246 KSLREQAKKMNIKSLRSNEDNKKASAEFRLAKVAFMTICCWFMAWTPYLTLSFLGIFSDR
                * *                  **                 * **    *

Bovine  281 SDFGPIFMTIPAFFAKTSAVYNPVIYIMMNKQFRNCM---VTTLCCGKNPLGDDEASTTV
JSR1    306 TWLTPMTSVWGAIFAKASACYNPIVYGISHPKYRAALHDKFPCLKCGSDSPKGDSASTVA
                *      * *** ** ***  *       *         * **      * ***

Bovine  338 SKTETSQ
JSR1    366 ESEKAGE
```

Figure S16: Sequence alignment of Bovine Rhodopsin (UniProt ID P02699) and Jumping Spider Rhodopsin 1 (UniProt ID B1B1U5).

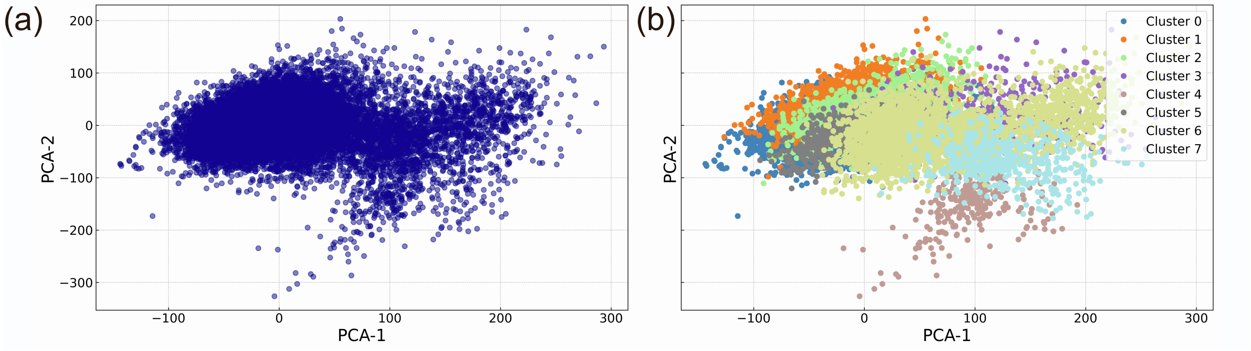

Figure S17: (a) PCA space obtained using the same data for the autoencoder training. (b) Points are colored according to the clustering performed in the autoencoder space of Figure 5 in the main text.

Table S1: Comparison of retinal dihedral angles for **9-cis** isomer: Molecular dynamics (MD) averages versus starting structure PDB 6I9K. Angles are reported in degrees (°). Key dihedrals indicating cis configurations (ϕ_4 and ϕ_6) are highlighted. SD is the standard deviation of the concatenated dataset.

**Dihedral Label**	**Atoms Involved**	**Description**	**6I9K (°)**	**MD Avg. ± SD (°)**	**Δ (°)**
ϕ_1	C5-C6-C7-C8	6-7 bond torsion	-61.4	-55.3 ± 12.1	6.1
ϕ_2	C6-C7-C8-C9	7-8 bond torsion	197.4	178.9 ± 10.0	-18.5
ϕ_3	C7-C8-C9-C10	8-9 bond torsion	180.9	180.6 ± 9.0	-0.3
ϕ_4	C8-C9-C10-C11	**9-10 bond**	-37.4	-26.2 ± 13.7	11.2
ϕ_5	C9-C10-C11-C12	10-11 bond torsion	193.0	184.4 ± 11.3	-8.6
ϕ_6	C10-C11-C12-C13	**11-12 bond**	159.5	168.4 ± 8.8	8.9
ϕ_7	C11-C12-C13-C14	12-13 bond torsion	181.6	175.1 ± 7.6	-6.5
ϕ_8	C12-C13-C14-C15	13-14 bond torsion	174.6	180.1 ± 8.3	5.5
ϕ_9	C13-C14-C15-N	14-15-Schiff base torsion	45.8	59.3 ± 16.1	13.5
χ_1	N-CA-CB-CG	Lysine sidechain torsion 1	96.6	79.6 ± 9.1	-17.0
χ_2	CA-CB-CG-CD	Lysine sidechain torsion 2	182.5	185.9 ± 5.3	3.4
χ_3	CB-CG-CD-CE	Lysine sidechain torsion 3	62.3	66.5 ± 7.2	4.2
χ_4	CG-CD-CE-NZ	Lysine sidechain torsion 4	-69.2	-71.1 ± 6.8	-1.8

Table S2: Comparison of retinal dihedral angles for the **11-*cis*** isomer: Molecular dynamics (MD) averages versus starting structure. Angles are reported in degrees (°). The key dihedral indicating the 11-*cis* configuration (ϕ_6) is highlighted.

**Dihedral Label**	**Atoms Involved**	**Description**	**Start. (°)**	**MD Avg. ± SD (°)**	**Δ (°)**
ϕ_1	C5-C6-C7-C8	6-7 bond torsion	-42.16	-33.67 ± 32.85	8.49
ϕ_2	C6-C7-C8-C9	7-8 bond torsion	183.73	184.66 ± 6.89	0.93
ϕ_3	C7-C8-C9-C10	8-9 bond torsion	141.99	139.47 ± 13.89	-2.52
ϕ_4	C8-C9-C10-C11	**9-10 bond**	154.88	167.35 ± 8.30	12.46
ϕ_5	C9-C10-C11-C12	10-11 bond torsion	194.23	185.00 ± 7.97	-9.22
ϕ_6	C10-C11-C12-C13	**11-12 bond**	-24.50	-10.55 ± 10.12	13.95
ϕ_7	C11-C12-C13-C14	12-13 bond torsion	167.91	158.26 ± 8.17	-9.65
ϕ_8	C12-C13-C14-C15	13-14 bond torsion	174.69	178.38 ± 9.96	3.68
ϕ_9	C13-C14-C15-N	14-15-Schiff base torsion	183.23	178.45 ± 8.36	-4.77
χ_1	N-CA-CB-CG	Lysine sidechain torsion 1	8.05	-0.12 ± 5.72	-8.18
χ_2	CA-CB-CG-CD	Lysine sidechain torsion 2	179.71	178.58 ± 6.32	-1.13
χ_3	CB-CG-CD-CE	Lysine sidechain torsion 3	189.04	206.63 ± 8.54	17.58
χ_4	CG-CD-CE-NZ	Lysine sidechain torsion 4	-44.97	-57.61 ± 8.25	-12.64

Table S3: Comparison of retinal dihedral angles for the **all-*trans*#1** isomer: Molecular dynamics (MD) averages versus starting structure. Angles are reported in degrees (°).

**Dihedral Label**	**Atoms Involved**	**Description**	**Start. (°)**	**MD Avg. ± SD (°)**	**Δ (°)**
ϕ_1	C5-C6-C7-C8	6-7 bond torsion	-10.03	-54.73 ± 20.81	-44.70
ϕ_2	C6-C7-C8-C9	7-8 bond torsion	206.24	183.14 ± 13.75	-23.09
ϕ_3	C7-C8-C9-C10	8-9 bond torsion	135.03	178.84 ± 9.00	43.80
ϕ_4	C8-C9-C10-C11	**9-10 bond**	216.68	203.04 ± 28.45	-13.64
ϕ_5	C9-C10-C11-C12	10-11 bond torsion	169.59	169.83 ± 17.50	0.23
ϕ_6	C10-C11-C12-C13	**11-12 bond**	203.80	191.91 ± 13.76	-11.88
ϕ_7	C11-C12-C13-C14	12-13 bond torsion	172.94	181.61 ± 7.83	8.66
ϕ_8	C12-C13-C14-C15	13-14 bond torsion	180.25	186.55 ± 11.13	6.29
ϕ_9	C13-C14-C15-N	14-15-Schiff base torsion	178.53	170.34 ± 21.15	-8.19
χ_1	N-CA-CB-CG	Lysine sidechain torsion 1	24.11	-49.12 ± 31.28	-73.23
χ_2	CA-CB-CG-CD	Lysine sidechain torsion 2	184.93	179.56 ± 6.66	-5.36
χ_3	CB-CG-CD-CE	Lysine sidechain torsion 3	179.95	-17.48 ± 93.28	-197.44
χ_4	CG-CD-CE-NZ	Lysine sidechain torsion 4	-77.44	-60.37 ± 7.24	17.06

Table S4: Comparison of retinal dihedral angles for an alternative **all-*trans*#2** isomer conformation: Molecular dynamics (MD) averages versus starting structure. Angles are reported in degrees (°).

**Dihedral Label**	**Atoms Involved**	**Description**	**Start. (°)**	**MD Avg. ± SD (°)**	**Δ (°)**
ϕ_1	C5-C6-C7-C8	6-7 bond torsion	152.10	132.25 ± 43.00	-19.84
ϕ_2	C6-C7-C8-C9	7-8 bond torsion	176.77	179.60 ± 9.94	2.83
ϕ_3	C7-C8-C9-C10	8-9 bond torsion	175.61	181.26 ± 8.49	5.64
ϕ_4	C8-C9-C10-C11	**9-10 bond**	221.91	183.37 ± 26.48	-38.53
ϕ_5	C9-C10-C11-C12	10-11 bond torsion	155.69	180.66 ± 15.98	24.97
ϕ_6	C10-C11-C12-C13	**11-12 bond**	198.92	183.30 ± 14.09	-15.62
ϕ_7	C11-C12-C13-C14	12-13 bond torsion	180.41	180.94 ± 7.27	0.53
ϕ_8	C12-C13-C14-C15	13-14 bond torsion	186.49	183.00 ± 12.00	-3.48
ϕ_9	C13-C14-C15-N	14-15-Schiff base torsion	176.20	159.50 ± 55.80	-16.70
χ_1	N-CA-CB-CG	Lysine sidechain torsion 1	-64.84	-57.89 ± 19.68	6.95
χ_2	CA-CB-CG-CD	Lysine sidechain torsion 2	171.89	178.64 ± 6.01	6.74
χ_3	CB-CG-CD-CE	Lysine sidechain torsion 3	-53.25	-40.66 ± 59.78	12.59
χ_4	CG-CD-CE-NZ	Lysine sidechain torsion 4	-58.00	-59.50 ± 6.49	-1.50

Table S5: Weights of each edge between pairs of residues along the communication paths.

Edge	9-*cis*	11-*cis*	all-*trans* #1	all-*trans* #2
Retinal → Trp290	2.83	1.60	3.07	0.91
Trp290 → Ala289	0.69	0.81	0.56	0.20
Ala289 → Met288	0.92	0.51	0.68	0.65
Met288 → Cys285	2.34	1.92	2.23	2.76
Cys285 → Cys284	0.38	0.45	0.38	0.66
Cys284 → Ile283	0.26	0.39	0.38	0.36
Ile283 → Thr282	0.49	0.45	0.40	0.50
Thr282 → Ala279	0.81	0.98	0.77	1.09
Ala279 → Ala276	1.42	1.46	1.34	1.67
Ala276 → Leu275	0.56	0.76	0.67	0.86
Leu275 → Glu272	3.31	3.11	2.46	2.82
Glu272 → Ala271	0.09	0.11	0.46	0.11
Ala271 → Ser270	0.18	0.11	0.14	0.09
Ser270 → Asn266	3.39	5.59	3.49	3.17
Asn266 → Asn263	1.47	0.71	0.60	0.68
Asn263 → Ser262	0.76	1.26	1.06	1.11
Ser262 → Ser259	1.30	1.25	1.28	1.34
Ser259 → Lys258	1.49	1.78	1.92	1.81
Path length (d_{min})	22.69	23.25	21.89	20.79
