# Supplementary material for: Mapping signal transduction in bistable jumping spider rhodopsin 1
Source: Biophys J. 2025 Nov 3;125(10):2287–97. doi: 10.1016/j.bpj.2025.10.040 (PMC13351664; doi:10.1016/j.bpj.2025.10.040)
Supplement: Document S2. Article plus supporting material [file mmc2.pdf]

Biophysical Journal
Article

Biophysical Society

Mapping signal transduction in bistable jumping spider rhodopsin 1

Flavio Costa,[1] Emanuele Telari,[2] Daniel Moreno-Rodríguez,[1] Simone Meloni,[3] Jógvan Magnus Haugaard Olsen,[4] Alberto Giacomello,[1] and Giovanni Di Muccio[1,5,*]

[1]Dipartimento di Ingegneria Meccanica e Aerospaziale, Sapienza Università di Roma, Rome, Italy; [2]Departament de Ciència de Materials i Química Física & Institut de Quimica Teòrica i Computacional (IQTCUB), Universitat de Barcelona, Barcelona, Spain; [3]Department of Chemical, Pharmaceutical and Agricultural Sciences, University of Ferrara, Ferrara, Italy; [4]DTU Chemistry, Technical University of Denmark, Kongens Lyngby, Denmark; and [5]NY-Masbic, Department of Life and Environmental Sciences, Marche Polytechnic University, Ancona, Italy

ABSTRACT G-protein-coupled receptors are key drug targets due to their role in cellular signaling. Among them, bistable rhodopsins such as the jumping spider rhodopsin 1 (JSR1), are promising for optogenetic applications, but their transduction mechanisms remain poorly understood. In this study, we used microsecond equilibrium molecular dynamics simulations, network analysis, and machine learning to investigate allosteric communication paths between the retinal chromophore and the intracellular G-protein-binding site in JSR1. We analyzed structural differences in three functional states with retinal chromophores in 9-*cis*, 11-*cis*, and all-*trans* configurations. Results revealed that Trp290 is crucial for transmitting the movements of the retinal after isomerization to the G-protein-binding site during JSR1 activation as well as residues along TM6 helix. Overall, these findings advance our understanding of bistable rhodopsins and their potential in light-driven technologies.

SIGNIFICANCE This research elucidates the molecular mechanisms underlying bistable jumping spider rhodopsin 1 activation, specifically identifying critical residues that transmit light-induced signals from the retinal chromophore to G-protein-binding sites. By revealing that Trp290 and TM6 helix residues serve as key intermediates in allosteric communication paths, this work provides essential structural insights for engineering improved optogenetic tools. The computational approach combining molecular dynamics simulations with machine learning offers a powerful framework for understanding GPCR signaling mechanisms. These findings have immediate implications for developing next-generation light-controllable proteins for therapeutic applications, including precision medicine approaches for treating neurological disorders, blindness, and other conditions requiring spatiotemporal control of cellular processes.

INTRODUCTION

G-protein-coupled receptors (GPCRs) are membrane proteins that play a key role in various processes such as sensory perception and inflammation, making them one of the most important pharmacological targets of drugs currently on the market (1,2). They exhibit a conserved protein architecture with seven transmembrane helices (TMs) connected by extracellular and intracellular loops (ECLs and ICLs, respectively), which can be divided into three functional domains (3): the orthosteric ligand-binding site (OBS) on the extracellular side, the connector, and the intracellular binding site (IBS) where signaling transducers such as G-proteins and β-arrestins bind (4,5). Structural rearrangements of OBS are transmitted to IBS, which, subsequently, undergoes structural changes that allow the transducers to bind the receptor, thus initiating the signal transduction cascade within the cell. This allosteric communication between OBS and IBS represents the molecular basis for the activation of GPCRs.

There are more than 800 GPCRs codified by the human genome (6), but the largest and well-studied class is represented by the rhodopsin-like family (7). This group includes rhodopsin, an ubiquitous protein expressed in several species whose defections are associated with vision and neurodegenerative pathologies (8). Here, the ligand inside the OBS is a retinal chromophore covalently bound to a lysine via a protonated Schiff base (PSB). Upon illumination, the retinal undergoes chemical modifications, that is, isomerization from *cis* (inactivated) to *trans* (activated) conformation,

Submitted June 26, 2025, and accepted for publication October 29, 2025.

*Correspondence: g.dimuccio@univpm.it

Editor: Ana Nicoleta Bondar.

https://doi.org/10.1016/j.bpj.2025.10.040

© 2025 The Author(s). Published by Elsevier Inc. on behalf of Biophysical Society.
This is an open access article under the CC BY license (http://creativecommons.org/licenses/by/4.0/).

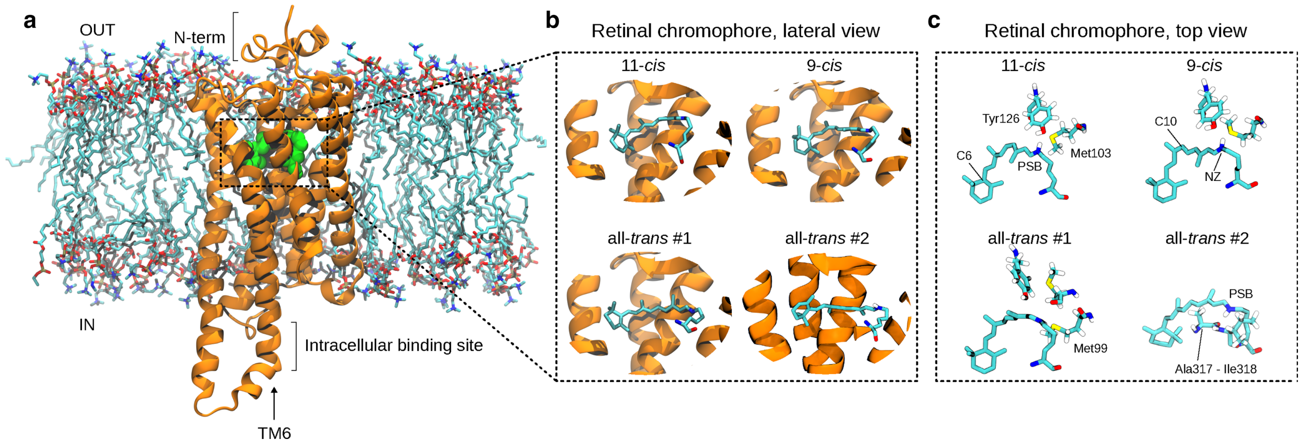

FIGURE 1 Jumping spider rhodopsin 1. (*a*) Illustration of the jumping spider rhodopsin 1 (JSR1) in complex retinal chromophore embedded in a lipid membrane model. The retinal chromophore inside the orthosteric ligand-binding site (*in green*) and the intracellular binding site with the transmembrane helix TM6 are highlighted. (*b–c*) Configurations of the retinal chromophore in the 11-*cis*, 9-*cis*, and all-*trans* #1/#2 systems, shown in side and top views. In the top views, residues within 4 Å of the PSB NH^+ are highlighted. Snapshots are MD representative frames; the set of contacting residues is illustrative of that frame and may vary during the trajectories. For 9-*cis*, the structure shows the PDB: 6I9K configuration, with the Tyr126 and Met103 coordinating the NH+.

which start the allosteric signal to IBS, inducing the receptor to structurally rearrange for activation. Previous experimental and computational studies have revealed the structural rearrangements that occur within the receptor during the activation process (9–13): slight modifications of 1.5–2 Å in the OBS and at the level of the intracellular ends of TM5 and TM7 helices; a significant outward/inward motion of 12–14 Å; and a rotation about 40°–50° of the intracellular side of helix TM6 in the IBS (14–19). However, the mechanism by which the signal is transmitted from the retinal chromophore to the IBS, especially to TM6 helix, is still unclear.

The isomerization of the retinal chromophore represents the initial step for the activation of all rhodopsins, but the photocycle can be different across the receptor family. In vertebrates, rhodopsin is characterized by a single stable state in the dark where the retinal Schiff base deprotonates along the photocycle, leading to the release of the chromophore and the bleaching of the sample (20). Invertebrates such as insects, spiders, or squids have a bistable rhodopsin that is characterized by two stable states associated with either light or dark. In this case, the Schiff base remains protonated throughout the entire photocycle (21,22), and the receptor can revert back to its original inactivated state through the absorption of another photon (light-triggered reversion), without the need of an enzymatic regeneration. This ability makes bistable rhodopsins good candidates for optogenetics and other biotechnological applications, as they can function as reversible photoswitches between their parent form and the photoproduct (23). However, although much is known about monostable rhodopsins, their bistable counterparts remain quite elusive.

In this context, jumping spider rhodopsin 1 (JSR1) from the spider *Hasarius adansoni* (24) emerges as a promising candidate for controlling cellular processes using light (Fig. 1 *a*). Its key advantage is being an animal receptor, presumably better suited for optogenetics in mammalian cells, especially in neuroscience research where precise neuronal control is critical. The crystal structure of JSR1 was recently solved with the retinal chromophore in the 9-*cis* configuration. This state, defined as “JSR1 isorhodopsin-1,” shows an absorbance maximum at 505 nm, which is blue-shifted compared with the 535-nm absorbance maximum of the native 11-*cis* retinal-bound form (25). However, both the 9-*cis* and the native 11-*cis* isoforms are bound to the inactive state of the protein, and both are still capable of activating to the all-*trans* form (19). Although the experimental structure lacks part of the N-terminus, the intracellular loop 3, and the C-terminus with TM8, the well-resolved part including transmembrane helices TM1 to TM7 highlights that JSR1 shares common structural features with the other members of the rhodopsin-like family.

In this work, we focus on the short- and long-range structural modifications induced by the retinal chromophore on the JSR1 structure as the protein changes at the level of the retinal binding site and the allosteric path coupling the retinal chromophore to the G-protein-binding site, respectively. We employed a combination of molecular dynamics (MD) simulations at the atomic scale, network analysis (26) to identify the long-range paths, and machine learning to classify the protein structural rearrangements across the JSR1 functional states (27). Our results show that Trp290 acts as a mechanical lever that transduces the movements caused by the isomerization of the retinal, thus propagating changes down to the intracellular side of rhodopsin, eventually leading to its activation. Moreover, we identify a series of residues on TM6 helix that act as important hubs in activation, including Ala289, Met288, Ile283, Thr282, Ala279,

Ala276, Leu275, and Glu272. These residues also play a crucial role in the activation of bovine rhodopsins. These insights provide a valuable basis for understanding the functionality of the bistable JSR1 and help establish future light-driven applications for the broader family of class A GPCRs.

MATERIALS AND METHODS

All-atom MD simulations

The JSR1 protein was modeled starting from the experimental structure solved by Varma et al. (25) (PDB: 6I9K) in the inactive state. The missing loops were modeled using SWISS-MODEL (28); using the APBS server (29), all aspartates and glutamates were predicted to be ionized, histidines His38, His244, and His335 to be in the δ state, and His36 and His50 in the ϵ state. As in the experimental structure, a disulfide bond between cysteines Cys123 and Cys200 was added. Four complexes were produced, hereafter named as "9-*cis*," "11-*cis*," all-*trans* #1 and all-*trans* #2, where the protein was bound to different configurations of retinal chromophore. In the first system, retinal was bound to the protein as in the experimental structure by Varma et al. (25) (PDB: 6I9K) (i.e., in the 9-*cis* configuration). In the 11-*cis* and all-*trans* #1 systems, the structures of JSR1 bound to retinal in 11-*cis* and all-*trans* configurations produced by Church et al. (30) were used. Finally, in the all-*trans* #2 system, retinal in all-*trans* configuration was complexed to the protein with the same orientation of that in the experimental structure recently solved by Tejero et al. (19). We performed a short (10-ns) steered molecular dynamics alignment, minimizing the root mean-square deviation (RMSD) of all-*trans* #1 to PDB: 9EPP over a selection comprising atoms C1–C15 (i.e., the orientation of the retinal backbone and the two methyl groups with respect to the β-ionone ring). Using the CHARMM Membrane Builder (31,32), all the complexes were embedded into a membrane of 260 POPC lipids, together with a solution of 0.15 NaCl and 41,603 TIP3P water molecules (33).

MD simulations were run with GROMACS 2023.3 (34) using the Amber ff14SB force field for the protein (35), the Lipid14 force field for the POPC bilayer (36), and TIP3P model for water (33), along with the parameters for the retinal chromophores derived by Church et al. (30). The temperature was maintained at 303.15 K by the Nosé-Hoover thermostat (37,38) with a damping coefficient of 1 ps $^{-1}$. Periodic boundary conditions were applied in all directions using a neighbor searching grid type and setting at 0.9 nm the cutoff distance for the short-range neighbor list. Electrostatic interactions were taken into account by using a fast and smooth particle mesh Ewald algorithm (39), with a 0.9-nm distance for the Coulomb cutoff. The integration time step was 2 fs. Three independent replicas per system were run for several microseconds in the NVT ensemble (more details in the next section). The stability of the proteins was assessed by their RMSD where only the Cα was considered and the reference conformation corresponded to that at the beginning of the dynamics. The trajectories were visually inspected using VMD 2.0 software (40).

Contact analysis

To identify the residue-residue and the residue-retinal interactions, semibinary contact maps (C_{ij}) were computed as truncated Gaussian kernels:

$$K\left(d_{ij}\right) = \begin{cases} 1, & d_{ij} \leq c \\ e^{-\left(d_{ij}^2 - c^2\right)/2\sigma^2}, & d_{ij} > c \end{cases}, \tag{1}$$

where d_{ij} is the distance between the side chain of the i-amino acid and either the side chain of the j amino acid or the j atom of the retinal chromophore; c is the cutoff distance set to 4.5 (41–44). The width σ of the Gaussian kernel was chosen so as to attain a negligibly small value of the kernel at $d_{ij} = 10$ Å. Specifically, we imposed $K(d_{cut}) = 10^{-5}$ attaining $\sigma = 1.38$. The final contact map was computed by averaging the value of the kernel over all the frames of the trajectory:

$$C_{ij} = \frac{1}{N_{\text{frames}}} \sum_{n=1}^{N_{\text{frames}}} K\left(d_{ij}(n)\right) \tag{2}$$

Network analysis

JSR1 with the retinal chromophores was represented as a graph where the weight assigned to the edges was as follows:

$$w_{ij} = -\,log\left(A_{ij}\right) = -\,log\left(C_{ij}M_{ij}\right), \tag{3}$$

with C_{ij} being the semibinary contact map and M_{ij} the mutual information matrix.

M_{ij} was computed as

$$M_{ij} = \frac{\sum_{d_i}\sum_{d_j} P\left(d_i, d_j\right) log \frac{P\left(d_i, d_j\right)}{P\left(d_i\right)P\left(d_j\right)}}{H_{ij}}, \tag{4}$$

where d_i and d_j are the displacement of the center of mass of the side chain of the i and j amino acids with respect to its average position, and H_{ij} is the Shannon entropy of those variables.

Dijkstra's algorithm (45) was used to compute the minimal paths between the retinal binding site (source) and the intracellular side of helix TM6 (sink). d_{min} corresponds to the lowest value computed from Eq. (3).

Machine learning classification

The structural classification was conducted by combining dimensionality reduction performed on appropriate structural descriptors with cluster analysis (27,46). The structural descriptors consisted of distance matrices derived from a selection of protein residues lying on the long-range communication path between retinal and the helix TM6: Lys258, Ser259, Ser262, Asn263, Asn266, Ser270, Ala271, Glu272, Leu275, Ala276, Ala279, Thr282, Ile283, Cys284, Cys285, Met288, Ala289, and Trp290. Each matrix was computed by calculating pairwise distances between the 129 atoms comprising this selection (excluding hydrogens), yielding a set of 129 $\times$ 129 symmetric matrices where the (i,j) element contains the distance (Å) between atoms i and j. These matrices were computed for 23,811 configurations obtained from equilibrium trajectories of each system, sampled every 1 ns.

The dimensionality reduction was performed via artificial neural networks using an autoencoder architecture (47). They are composed by encoder and decoder, which share a mirrored structure and are built by a sequence of repeated building blocks. In the encoder, such building blocks are formed by a convolutional layer, a batch normalization layer, and a max pooling layer. In the decoder, the convolutional layer is replaced by a deconvolutional one and the max pooling by an upscaling. Before the bottleneck, the channels of the last convolutional layer are flattened and fed to a fully connected layer. Afterward, they are reshaped and fed into the decoder. These network architectures perform nonlinear dimensionality reduction by learning to reconstruct input data at the output while being forced through a compressed representation at the bottleneck. The bottleneck thus forms a low-dimensional representation of the input with dimensionality determined by the number of nodes in that layer. The autoencoders were implemented in PyTorch (48), employing 2D convolutional layers in the encoder and 2D transposed convolution (deconvolutional) layers in the decoder, which are well suited for processing matrix-like data such as

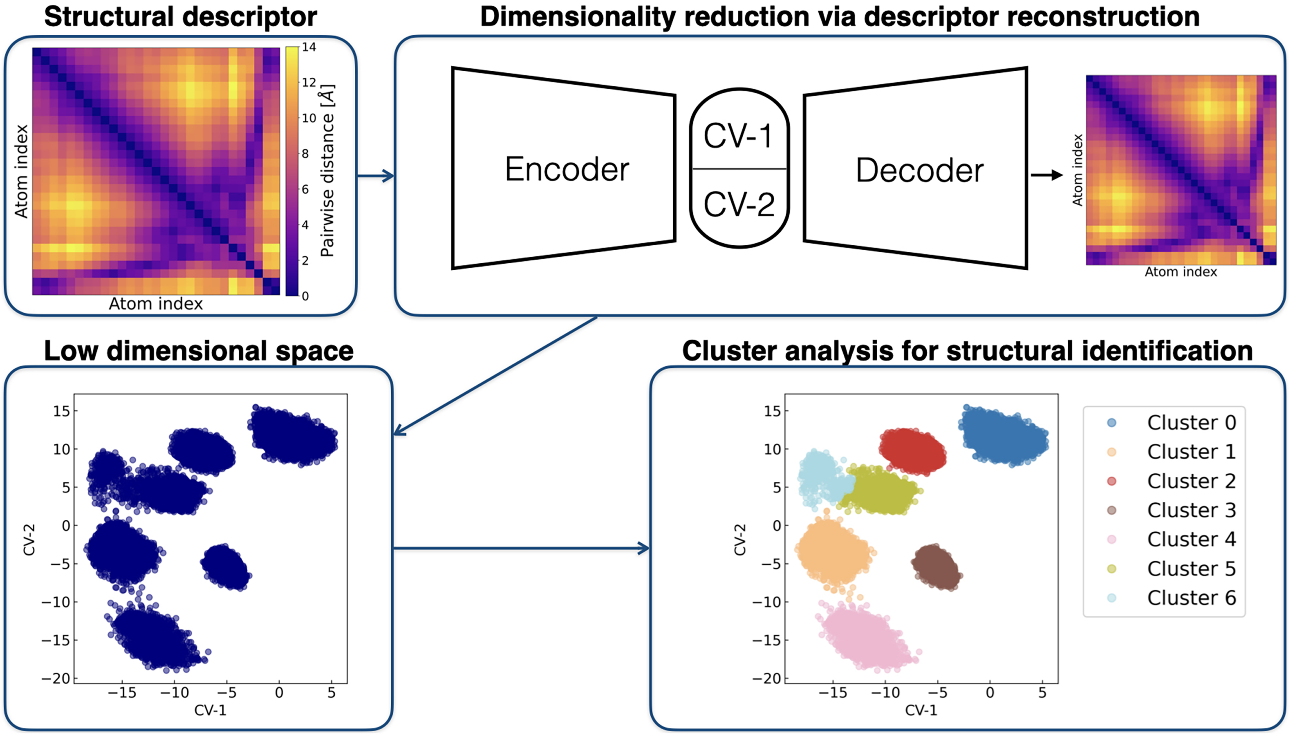

FIGURE 2 Scheme of the machine learning classification. Distance matrices computed from atomic selections along the retinal-TM6 communication pathway serve as structural descriptors (*top left*). These are input to an autoencoder neural network, which reduces dimensionality by learning a compressed representation in a two-dimensional latent space (CV-1, CV-2) and reconstructs the original descriptor (*top center and right*). The latent space representations (*bottom left*) are analyzed through agglomerative clustering to identify structurally distinct conformational states (*bottom right*).

distance matrices (49,50). In Fig. S1, there is a detailed description of the network as printed by torchsummary. The bottleneck size was fixed at 2 to obtain two-dimensional representations of the different atom selections. The general scheme of the protocol is resumed in Fig. 2.

Training was carried out by minimizing the mean-square error loss computed between inputs and reconstructed outputs with the Adam optimizer (51). The training data set comprised 23,811 matrices. Data were shuffled and split into training and validation sets, with the former containing 80% of the data set and the latter the remaining 20%. A batch size of 64 was employed. Before training, data were normalized by setting the minimum and maximum values of every feature to 0 and 1, respectively. The initial learning rate was set to 0.005 with a multistep scheduler that halved values at epochs 30 and 100. Training convergence was assessed using early stopping with a patience of 5 epochs and a minimum improvement threshold of 5×10^{-5} on validation loss (Figs. S2–S4).

The cluster analysis was then performed on the low-dimensional representations obtained from the autoencoders using agglomerative clustering implemented with the scikit-learn Python library (52). Specifically, Ward's linkage criterion (53) was employed in combination with the Euclidean distance metric. To assess clustering quality and determine the optimal number of clusters, three standard evaluation metrics were used: 1) the average silhouette score, where the silhouette score for a point i is defined as

$$s(i) = \frac{b(i) - a(i)}{\max\{a(i), b(i)\}}, \tag{5}$$

where $a(i)$ is the average distance to all other points in the same cluster, and $b(i)$ is the minimum average distance to points in any other cluster. The overall score is the average of $s(i)$ over all data points. Higher values (closer to 1) indicate better-defined clusters. 2) The Davies-Bouldin Score is computed as

$$\mathrm{DB} = \frac{1}{k}\sum_{i=1}^{k} \max_{j \neq i}\left(\frac{\sigma_i + \sigma_j}{d_{ij}}\right), \tag{6}$$

where σ_i and σ_j are the average distances of points in clusters i and j to their respective centroids, and d_{ij} is the Euclidean distance between the centroids of clusters i and j. Lower values indicate better separation between clusters. 3) The Calinski-Harabasz Score, also known as the variance ratio criterion, is defined as

$$\mathrm{CH} = \frac{\mathrm{Tr}(B_k)}{\mathrm{Tr}(W_k)} \frac{n - k}{k - 1}, \tag{7}$$

where $\mathrm{Tr}(B_k)$ is the trace of the between-cluster dispersion matrix, $\mathrm{Tr}(W_k)$ is the trace of the within-cluster dispersion matrix, n is the number of samples, and k is the number of clusters. Higher values indicate more distinct and well-separated clusters. Their values are reported in Fig. S5 for a number of clusters ranging from 2 to 15.

RESULTS AND DISCUSSION

The computational complexes of JSR1 with different conformations of retinal chromophores were produced (Fig. 1 *b*) as follows. The first simulated system, hereafter named as "9-*cis*," was produced starting from the complex solved by Varma et al. (25), which is characterized by both the protein and the chromophore in the inactive state (i.e., JSR1

TABLE 1 Length of each MD simulation and reference point after which the systems reached the convergence monitored via RMSD calculations

System	# Replica	Total time	Convergence
9-*cis*	Rep #1	1500 ns	After 300 ns
	Rep #2	1500 ns	After 250 ns
	Rep #3	1500 ns	After 750 ns
11-*cis*	Rep #1	1000 ns	After 100 ns
	Rep #2	1000 ns	After 100 ns
	Rep #3	1000 ns	After 100 ns
all-*trans* #1	Rep #1	2000 ns	After 950 ns
	Rep #2	2000 ns	After 900 ns
	Rep #3	2000 ns	After 750 ns
all-*trans* #2	Rep #1	2000 ns	After 900 ns
	Rep #2	2000 ns	After 500 ns
	Rep #3	2000 ns	After 1300 ns

isorhodopsin-1 with 9-*cis* retinal). Since no other structures had been solved when the present work started, the same protein structure was used to produce the systems “11-*cis*” and “all-*trans* #1” where the corresponding retinal configurations were taken from Church et al. (30). Thus, these systems were characterized by the protein in the inactive state and the retinal chromophore either in the inactive (11-*cis*) or in the active conformation (all-*trans*). However, at the time of writing this paper, we became aware of the work by Tejero et al. (19) with new experimental structures of JSR1 in the active state. These structures are characterized by retinal in all-*trans* conformation, which was oriented in the binding pocket differently from that in the complex by Church et al. (30). Thus, we studied another system named “all-*trans* #2” which is characterized by the same protein structure used for the other models (the inactive state taken from Varma et al. (25)) but with the retinal in the new active configuration as in Tejero et al. (19). The rationale for employing the inactive protein in complex with the active retinal (all-*trans*) was to investigate the short- and long-range structural rearrangements triggered by the active chromophore (all-*trans*) within an otherwise inactive receptor, thereby capturing early events in the activation process. Consequently, MD simulations of JSR1 with retinal chromophores in 9-*cis*, 11-*cis*, all-*trans* #1, and all-*trans* #2 configurations were run for several microseconds (Table 1). Considering that each replica reached a steady state after a different number of steps (Table 1; Fig. S6), cumulative times of dynamics of 3.2 μs for 9-*cis*, 2.7 μs for 11-*cis*, 3.4 μs for all-*trans* #1, and 3.3 μs for all-*trans* #2 systems were used for collecting data. As an additional check on retinal dynamics, we analyzed all retinal dihedral angles—nine along the polyene chain and four from the lysine side chain—across the three independent 9-*cis* simulation replicas. The full time series for each dihedral in each replica and summary histograms is shown in Figs. S7 and S8, respectively, and averages are presented in Table S1. The retinal conformation remained stable throughout all simulations. In particular, the key dihedral C8–C9–C10–C11 maintained a *cis* value of $-26.2° \pm 13.7°$, consistent with the starting crystal structure PDB: 6I9K ($-37.4°$). Similar plots for all simulated systems listed in Table 1 are shown in Figs. S9–S14.

Short-range protein structural modifications induced by retinal

To assess how different retinal isomers influence the short-range structural dynamics of the binding pocket, we performed a contact analysis across 9-*cis*, 11-*cis*, and two independent all-*trans* configurations (Table 2; Fig. 3). Each chromophore was found to engage with a conserved network of hydrophobic and aromatic residues, with key contacts involving Ile57, Met99, Leu100, Met103, Met107, Tyr126, Ser131, Ser199, Thr201, Ile202, Tyr218, Ala219, Val222, Tyr223, Trp290, Tyr293, Leu294, Ala317, Ile318, Phe319, Ala320, Ala322, Ser323, and Ala324—residues that appear consistently across all isomeric states. Among these, Met103 and Tyr293 are known to stabilize the retinal within the pocket (25,30,54).

The contact profile of 11-*cis* also includes unique interactions with Gly134, Ser135, Ile138, and Cys325, absent in the 9-*cis* and all-*trans* states, whereas 9-*cis* uniquely features Gly130 (Fig. S15). The all-*trans* forms differ slightly: Leu215, Trp286, and Ser297 are specific to all-*trans* #1,

TABLE 2 Residue contacts with retinal in different isomeric states

Residue name	9-*cis*	11-*cis*	All-*trans* #1	All-*trans* #2
Trp	286, **290**	286, **290**	286, **290**	**290**
Phe	**319**	**319**	**319**	**319**
Ile	**57**, 60, **202**, **318**	**57**, 60, **138**, **202**, **318**	**57**, **202**, **318**	**57**, 60, **202**, **318**
Leu	**100**, **215**, **294**	**100**, **215**, **294**	**100**, **215**, **294**	**100**, **294**
Val	**222**	**222**	**222**	**222**
Met	**99**, **103**, **107**	**99**, **103**, **107**	**99**, **103**, **107**	**99**, **103**, **107**
Ala	**219**, **317**, **320**, **322**, **324**	**219**, **317**, **320**, **322**, **324**	**219**, **317**, **320**, **322**, **324**	**219**, **317**, **320**, **322**, **324**
Gly	**127**, **130**	**127**, 134	–	**130**
Ser	**131**, **199**, **297**, **323**	**131**, **135**, **199**, **297**, **323**	**131**, **199**, **297**, **323**	**131**, **199**, **323**
Thr	**201**	**201**	**201**	**201**
Tyr	**126**, **191**, **218**, 223, **293**	**126**, **191**, **218**, 223, **293**	**126**, **218**, 223, **293**	**126**, **218**, 223, **293**
Cys	–	325	–	–

Residues interacting in more than 80% of the configurations are shown in bold.

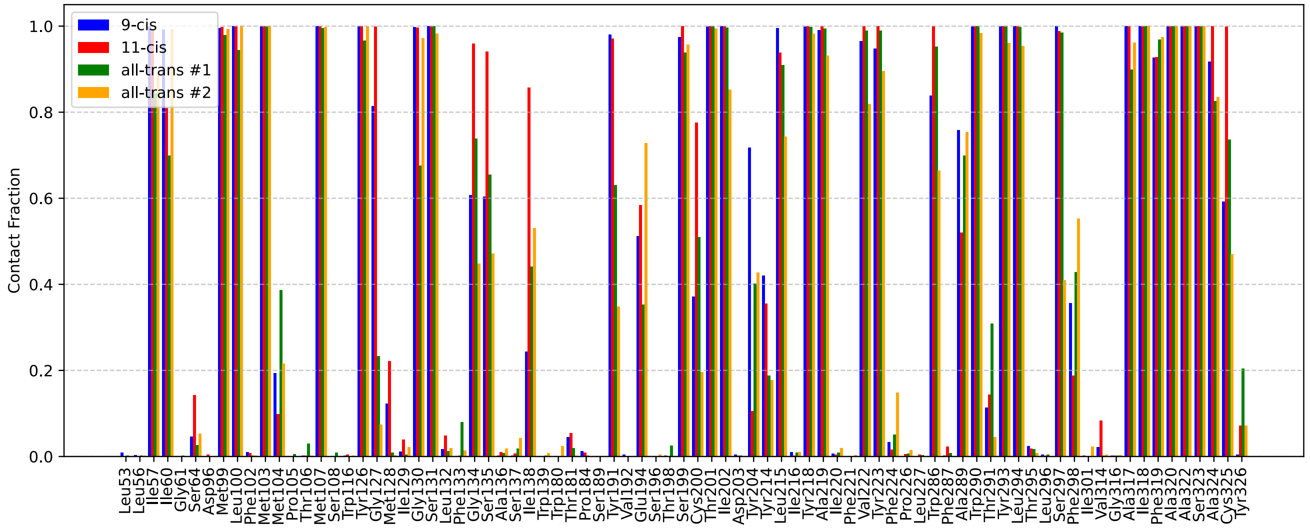

FIGURE 3 Protein residues interacting with retinal in all conformations. The cutoff distance used to identify the interactions was 4.5 Å. A contact fraction equal to 1 indicates that the interaction persists during the whole trajectory.

whereas Ile60 and Gly130 are only present in all-*trans* #2. Interestingly, no residues are uniquely shared between the two all-*trans* configurations without also appearing in at least one of the *cis* forms, underscoring the higher structural variability of the active-like retinal within an inactive protein scaffold.

This observation is further supported by the analysis of hydrogen-bonding distances. Recent work by Tejero et al. (19) showed that Tyr126 stabilizes the PSB in the 9-*cis* conformation via a short 2.9-Å contact with the nitrogen on the retinal. In our simulations, this distance was 3.2 ± 0.2 Å in 9-*cis* and 3.1 ± 0.2 Å in 11-*cis*, but it increased to 4.2 ± 0.3 Å and 3.7 ± 0.4 Å in all-*trans* #1 and #2, respectively. A similar trend was observed for Ser199, with PSB distances of 4.1 ± 0.2 Å (9-*cis*), 3.8 ± 0.3 Å (11-*cis*), 5.5 ± 0.4 Å (all-*trans* #1), and 4.6 ± 0.5 Å (all-*trans* #2), compared with crystallographic values of 3.9 Å (inactive) and 5.7 Å (active) (19,25).

Additionally, we found that several JSR1 residues involved in retinal binding correspond to pathogenic mutation sites in bovine rhodopsin (e.g., Gly127, Ile138, Tyr191, Ser199, Thr201, Tyr218), further supporting their functional importance (55).

Overall, the contact analysis reveals that the binding pocket undergoes state-specific rearrangements upon isomerization, with 11-*cis* exhibiting the richest and most diverse interaction profile, and all-*trans* states showing less extensive, though still functionally significant, contacts. Consistent with this picture, the dihedral summaries (Tables S1–S4) indicate that hallmark torsions retain their expected *cis* signatures in the *cis* ensembles; for example, in 9-*cis* the key C8–C9–C10–C11 torsion (ϕ_4) is narrowly centered at $-26.2° \pm 13.7°$ and C10–C11–C12–C13 (ϕ_6) at $168.4° \pm 8.8°$; in 11-*cis*, ϕ_6 shifts to a *cis*-like value of $-10.6° \pm 10.1°$—whereas most remaining polyene angles occupy single, relatively tight wells. In contrast, the all-*trans* forms drive several polyene torsions toward ~180° with broader distributions (e.g., $\phi_4 = 203.0° \pm 28.5°$ in all-*trans* #1 and $183.4° \pm 26.5°$ in all-*trans* #2) and increase flexibility at the ionone-polyene junction and the Schiff-base linkage (e.g., ϕ_9 SDs of 21.2° in all-*trans* #1 and 55.8° in all-*trans* #2). Notably, the lysine side chain becomes markedly more labile in the active-like ensembles, with χ_3 showing very large fluctuations (SD 93.3° in all-*trans* #1; 59.8° in all-*trans* #2), consistent with the weakened PSB-stabilizing contacts (longer Tyr126/Ser199 distances) and with the Trp290 rotation/TM6 rearrangement isolated by our ML classification (see next sections). Thus, beyond the *cis*/*trans* assignment, the isomer-dependent redistribution and broadening of specific retinal torsions—especially around C13–C15 and the β-ionone ring—map onto the state-specific interaction patterns in Fig. 3, providing a mechanistic link between chromophore conformational ensembles and pocket remodeling.

Long-range communication path between retinal and the G-protein-binding site

To predict the long-range communication mechanism coupling the retinal chromophore to the G-protein-binding site (i.e., the molecular basis of JSR1 activation), a network analysis was employed. First, the complexes were represented as networks in which nodes coincide with the protein amino acids and edges with the interactions between pairs. Then, a weight expressed as $w_{ij} = -log(A_{ij}) = -log(C_{ij}M_{ij})$ was assigned to each edge, which quantifies the communication path in terms of contacts between amino acids and correlations of their motion, computed from MD runs. Dijkstra's algorithm (45) was then used to determine the shortest paths, that is, the most effective communication

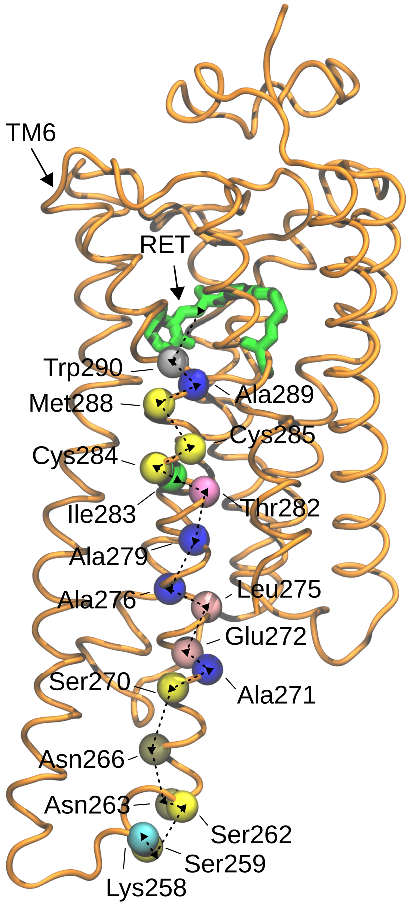

FIGURE 4 Communication path between the retinal chromophore and helix TM6 in all-*trans* systems. Retinal is shown in green licorice, whereas the Cα of residues along the paths are colored by name and represented in VdW.

routes, between the retinal binding pocket (source) and the IBS localized at the end of TM6 helix in the inactive configuration (sink). The outward rotation of the sink site was shown to form the G-protein-binding cavity (4,12).

In the systems all-*trans* #1 and #2, the path was found to jump from retinal to the C-terminal side of TM6 at the level of Trp290 (Fig. 4). Then, the path moves down to reach the intracellular side of TM6 helix involving Ala289, Met288, Cys285, Cys284, Ile283, Thr282, Ala279, Ala276, Leu275, Glu272, Ser270, Asn266, Asn263, Ser262, Ser259, and Lys258. Interestingly, mutations of bovine rhodopsin known to affect the activation of the protein correspond to residues on JSR1 identified along the path. They are Cys264 (56), Ile263 (56), Val258 (57), Met257 (58), Val254 (59), Thr251 (60), Val250 (61), and Glu247 (12), which correspond to Ala289, Met288, Ile283, Thr282, Ala279, Ala276, Leu275, and Glu272, respectively, in JSR1 (Fig. S16). This evidence supports the robustness of our findings.

The pathlength (d_{min}), which describes the efficiency of the coupling mechanism and results from the sum of the weights of each edge, was $d_{min} = 21.89$ for all-*trans* #1 and $d_{min} = 20.79$ for all-*trans* #2. Considering the logarithmic nature of this metric, a unit difference in d_{min} corresponds to an order-of-magnitude change in communication efficiency. Thus, the allosteric path is more efficient in all-*trans* #2 than in all-*trans* #1.

As a control, the same analysis was carried out in the 9-*cis* and 11-*cis* complexes. From a qualitative perspective, both systems share the same route coupling the retinal to TM6 helix, involving identical protein residues. However, their path lengths differ from those of the all-*trans* systems, with $d_{min} = 22.69$ in 9-*cis* and $d_{min} = 23.25$ in 11-*cis*, indicating that the path has a weaker communication efficiency in the *cis* systems. Interestingly, the comparison of the edge weights between pairs of residues along the paths (Table S1) revealed that the bottleneck reducing communication efficiency in the *cis* systems compared with the all-*trans* is localized at the bending region of TM6. Indeed, 11-*cis* shows the highest weight value at the Ser270→ Asn266 step (5.59) among all the systems, whereas 9-*cis* shows a peak at Asn266→ Asn263 (1.47 versus 0.71 in 11-*cis*, 0.60 in all-*trans* #1, and 0.68 in all-*trans* #2).

Overall, these results indicate that communication between the retinal chromophore and the G-protein-binding site, defined by TM6 helix, involves Trp290 as a key bridge between these regions, together with several other residues on TM6. Moreover, the path did not change across the systems; however, their lengths suggest the following trend in communication efficiency: all-*trans* #2 > all-*trans* #1 > 9-*cis* > 11-*cis*, with approximately one order of magnitude efficiency drop at each inequality. In this context, all-*trans* #2 appears to adopt the most favorable active pose of the retinal within the binding pocket to efficiently activate the protein. Indeed, although the retinal aldehyde tail and the nearby methyl group stabilize Trp290, contributing to maintaining the stable position of its side chain, the second retinal methyl group that is closer to the β-ionone ring is able to interact better with the indole ring of Trp290, improving the efficiency of the signal propagation toward TM6 helix.

Autoencoder-based structural classification of the Retinal-TM6 signaling path

To predict the structural rearrangements of the residues along the previously described allosteric path between retinal and TM6 helix across the JSR1 functional states, a machine learning structural classification was adopted. All the available MD configurations were classified in eight structural clusters, which were further grouped into three distinct families (Fig. 5). The comparison with principal components analysis illustrates well the capability of the nonlinear technique (i.e., the autoencoder-based classification) to discriminate structural patterns while keeping the dimensionality of the representation low (Fig. S17). The first family contains cluster 5, characterized by a downward

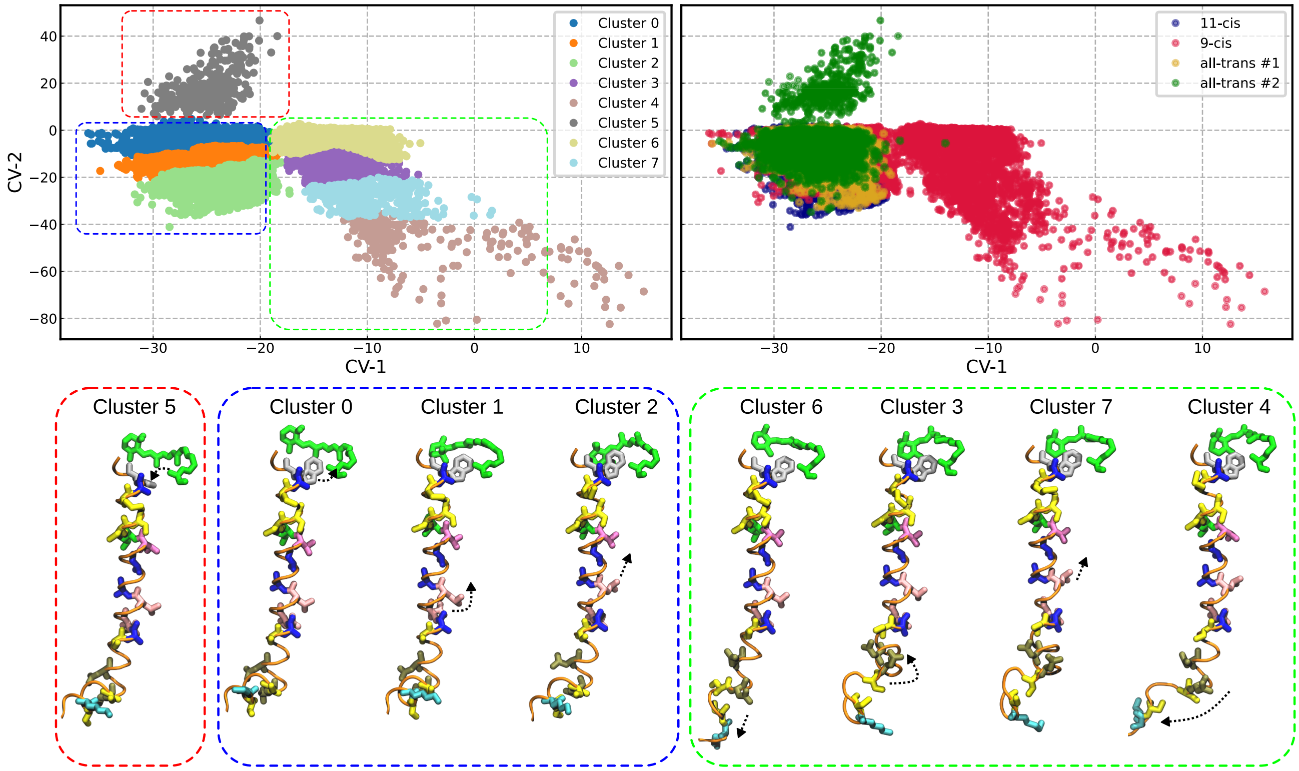

FIGURE 5 Diagram depicting the structural transitions of the residues along the path where the most representative structures of each cluster are reported. Dashed squares highlight the main cluster families, and black arrows describe the structural difference of that cluster with respect to the others. Retinal is in green, and the residues along the path are colored by individual amino acids.

rotation of Trp290. The second family encompasses clusters 0–2, showing an upward displacement of Glu272 and Leu275. Finally, the third family comprises clusters 6, 3, 7, and 4, where the primary structural differences are localized at the bottom of TM6, specifically a clockwise rotation of Lys258, Ser259, Ser262, and Asn266, accompanied by a slight contraction of the helix toward the upper region. The most significant findings emerge from cluster 5 and cluster 4: cluster 5 exhibits a rotation of Trp290, whereas cluster 4 displays a prominent bending of the end side of TM6 together with a clockwise rotation. By comparing the structural differences of these clusters with the corresponding systems they appear in, two distinct patterns emerge: the rotation of Trp290, observed exclusively in the all-*trans* #2 system, and the bending coupled to rotation of the bottom side of TM6 found in the 9-*cis* system. Notably, Trp290, which has been experimentally demonstrated to play a crucial role in the activation of vertebrate rhodopsins (1,14,62,63), is found to rotate only in all-*trans* #2, which represents the most realistic active pose of retinal in the binding pocket. This observation can be interpreted as the initial step in protein activation, where Trp290 transduces the isomerization of the retinal and transmits this conformational change to the rest of the protein.

Inspection of the retinal dihedral time series for frames assigned to cluster 5 (Fig. S13) reveals a transient 14-*cis* geometry at the C14–C15 double bond sampled in replicate 1 of all-*trans* #2. Although this feature could be construed as a classical force field artifact, it arose spontaneously and was not reproduced elsewhere; together with the two replicas that remain all-*trans*, this indicates that the torsion is on average stable and supports its interpretation as a short-lived excursion along the active-like pathway rather than a preparation bias. Consistent with this, the Schiff-base torsion (C13–C14–C15–NZ) displays the largest—yet still weak—Pearson $|r| \leq 0.15$ correlation with Trp290 rotation across 9-*cis*, 11-*cis*, and all-*trans* #1.

As a complementary quantitative check of chromophore-protein coupling, we computed Pearson correlations between each retinal dihedral and the Trp290 CA–CB–CG–CD2 dihedral over pooled equilibrium segments (Fig. 6; complete time series and distributions in Figs. S7–S14). Across all data sets, correlations remain modest (typically $|r| \lessapprox 0.10$), with slightly elevated values near C13–C14/C14–C15 and for the β-ionone ring, most evident when all-*trans* #2 is included. Together with the autoencoder-based classification (Fig. 5), which isolates a Trp290-rotated family unique to all-*trans* #2, these trends support all-*trans* #2 as the activating arrangement: retinal isomerization perturbs the pocket to favor Trp290 rotation and TM6 rearrangement, with coupling distributed over several torsions rather than dominated by a single dihedral.

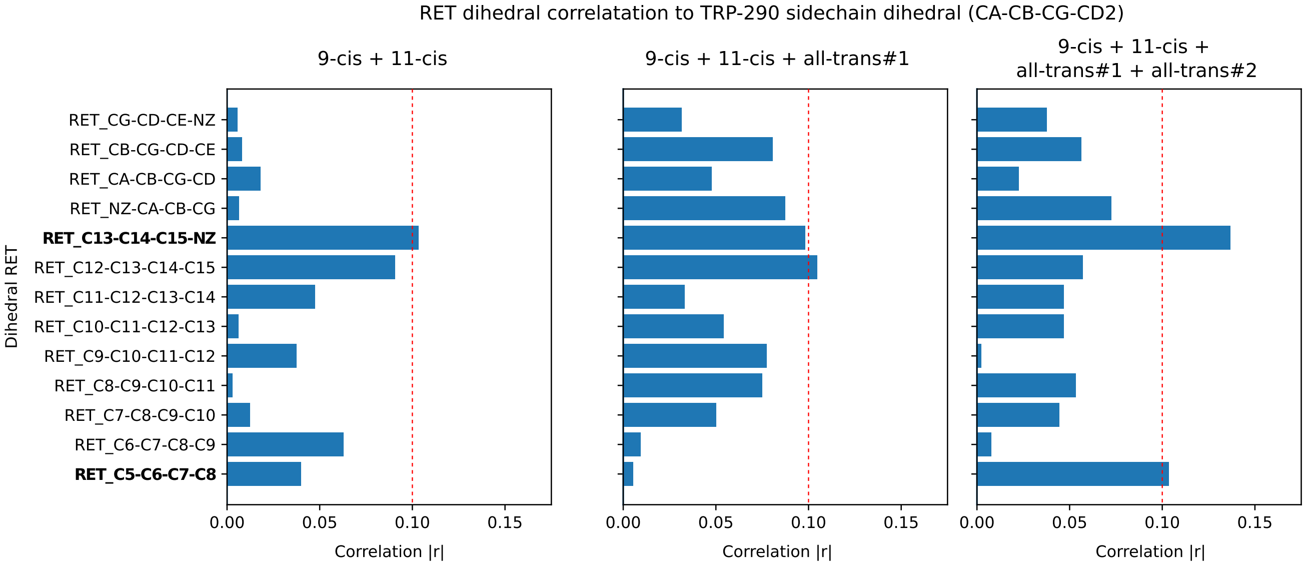

FIGURE 6 Bar plots of the absolute Pearson correlation coefficients, $|r|$, between each retinal dihedral and the Trp290 side-chain dihedral (CA–CB–CG–CD2). The three columns represent correlations computed over different simulation sets: left, *9-cis* and *11-cis*; middle, plus all-*trans* #1; right, the full data set including all-*trans* #2. Complete time series and distributions for all dihedrals are shown in Figs. S7–S14. Across all sets, correlations are modest (typically $|r| \lessapprox 0.1$), indicating very weak linear co-variation between Trp290 rotation and any single retinal torsion. Nevertheless, torsions around C13–C14/C14-C15 and the β-ionone ring orientation (C5-C6-C7-C8) show slightly higher $|r|$, most evident when including the all-*trans* #2 data set.

CONCLUSION

In this work, we investigated the molecular mechanisms underlying the activation of JSR1 by integrating microsecond-scale MD simulations, network analysis, and machine learning-based structural classification. By comparing four JSR1 systems complexed with different isomers of the retinal chromophore, we found that the all-*trans* #2 configuration appears to represent the most favorable active pose within the binding pocket. This configuration exhibited the most efficient allosteric communication between the retinal and the intracellular G-protein-binding site, as indicated by the shortest network pathlength ($d_{min} = 20.79$), compared with all-*trans* #1 ($d_{min} = 21.89$), 9-*cis* ($d_{min} = 22.69$), and 11-*cis* ($d_{min} = 23.25$).

The network analysis has been widely used to identify allosteric paths between two protein regions (26). Here, it was combined with machine learning in order to identify the conformational poses of the residues along the paths in the presence of different retinal isomers. Two different stages of the activation process were found: 1) the rotation of Trp290 and 2) the bending of TM6 characterized by a clockwise rotation of Lys258, Ser259, Ser262, and Asn266, accompanied by a slight contraction of the helix toward the upper region of TM6. These results agree with previous evidence on different GPCR classes (4,12). This combined method provided a powerful framework for studying allostery in JSR1, which can be applied to other GPCR systems, and, in general, to membrane proteins.

Our findings identify Trp290 as a key molecular switch in the JSR1 activation process. Located at the junction between the chromophore and TM6 helix, Trp290 acts as a primary sensor of retinal isomerization. Its rotation—observed exclusively in the all-*trans* #2 system— marks the initial step of activation, transmitting structural rearrangements to the intracellular portion of TM6. This result is consistent with previous studies on vertebrate rhodopsins, where Trp290 has been experimentally validated as essential for activation (1,14,62). Thus, it represents a strategic residue that can be mutated to tune the activation of JSR1: nonconservative mutations (e.g., to alanine), which remove the bulky indole ring crucial for the interaction with retinal, are expected to abolish the activation.

In conclusion, this study offers a comprehensive molecular-level view of JSR1 signal transduction, highlighting several crucial residues that can be targeted by mutagenesis to regulate JSR1 function. It underscores the functional relevance of the all-*trans* #2 retinal configuration and elucidates the structural role of Trp290 as a mechanistic bridge between chromophore isomerization and intracellular signaling. Moreover, it introduces a generalizable strategy that couples network analysis with machine learning to characterize allosteric communication in complex biomolecular systems.

ACKNOWLEDGMENTS

F.C. acknowledges the financial support by the Italian Ministry for Education, University and Research (MIUR) through the "Framework per l'Attrazione e il Rafforzamento delle Eccellenze per la Ricerca in Italia (FARE)" scheme, grant SERENA n. R18XYKRW7J. The authors acknowledge EuroHPC for awarding them access to MareNostrum5 (project ID

ehpc13) at Barcelona Supercomputing Center, Spain. GDM acknowledge the National Biodiversity Future Center (NBFC), funded by the Italian National Recovery and Resilience Plan (PNRR), Project Code CN00000033, CUP I33C22001300007, under the European Union's NextGenerationEU program.

AUTHOR CONTRIBUTIONS

F.C. performed the research (simulations, contact analysis, and network analysis) and wrote the paper; E.T. performed the research (machine learning classification) and wrote the paper; D.M.-R. supported the research; S.M. supervised the research; A.G. supervised the research and reviewed the paper; J.M.H.O. supervised the research; G.D.M. designed and performed the research (system setup and simulations) and reviewed the original draft of the paper. All authors gave approval to the final version of the manuscript.

DECLARATION OF INTERESTS

The authors declare no competing interests.

SUPPORTING MATERIAL

Supporting material can be found online at https://doi.org/10.1016/j.bpj.2025.10.040.

REFERENCES

1. Rosenbaum, D. M., S. G. F. Rasmussen, and B. K. Kobilka. 2009. The structure and function of G-protein-coupled receptors. *Nature.* 459:356–363.
2. Yang, D., Q. Zhou, …, M. W. Wang. 2021. G protein-coupled receptors: structure-and function-based drug discovery. *Signal Transduct. Targeted Ther.* 6:7.
3. Hilger, D., M. Masureel, and B. K. Kobilka. 2018. Structure and dynamics of GPCR signaling complexes. *Nat. Struct. Mol. Biol.* 25:4–12.
4. Weis, W. I., and B. K. Kobilka. 2018. The molecular basis of G protein–coupled receptor activation. *Annu. Rev. Biochem.* 87:897–919.
5. Latorraca, N. R., J. K. Wang, …, R. O. Dror. 2018. Molecular mechanism of GPCR-mediated arrestin activation. *Nature.* 557:452–456.
6. Fredriksson, R., M. C. Lagerström, …, H. B. Schiöth. 2003. The G-protein-coupled receptors in the human genome form five main families. Phylogenetic analysis, paralogon groups, and fingerprints. *Mol. Pharmacol.* 63:1256–1272.
7. Katritch, V., V. Cherezov, and R. C. Stevens. 2013. Structure-function of the G protein–coupled receptor superfamily. *Annu. Rev. Pharmacol. Toxicol.* 53:531–556.
8. Lenahan, C., R. Sanghavi, …, J. H. Zhang. 2020. Rhodopsin: a potential biomarker for neurodegenerative diseases. *Front. Neurosci.* 14:326.
9. Schönegge, A.-M., J. Gallion, …, M. Bouvier. 2017. Evolutionary action and structural basis of the allosteric switch controlling β2AR functional selectivity. *Nat. Commun.* 8:2169.
10. Filipek, S. 2019. Molecular switches in GPCRs. *Curr. Opin. Struct. Biol.* 55:114–120.
11. Chen, Y., O. Fleetwood, …, L. Delemotte. 2021. Allosteric effect of nanobody binding on ligand-specific active states of the β2 adrenergic receptor. *J. Chem. Inf. Model.* 61:6024–6037.
12. Smith, S. O. 2023. Mechanism of activation of the visual receptor rhodopsin. *Annu. Rev. Biophys.* 52:301–317.
13. D'Amore, V. M., P. Conflitti, …, V. Limongelli. 2024. Minute-timescale free-energy calculations reveal a pseudo-active state in the adenosine A2A receptor activation mechanism. *Chem.* 10:3678–3698.
14. Venkatakrishnan, A. J., X. Deupi, …, M. M. Babu. 2013. Molecular signatures of G-protein-coupled receptors. *Nature.* 494:185–194.
15. Manglik, A., and A. C. Kruse. 2017. Structural basis for G protein-coupled receptor activation. *Biochemistry.* 56:5628–5634.
16. Hauser, A. S., A. J. Kooistra, …, D. E. Gloriam. 2021. GPCR activation mechanisms across classes and macro/microscales. *Nat. Struct. Mol. Biol.* 28:879–888.
17. Rosenbaum, D. M., C. Zhang, …, B. K. Kobilka. 2011. Structure and function of an irreversible agonist-β2 adrenoceptor complex. *Nature.* 469:236–240.
18. Rasmussen, S. G. F., H.-J. Choi, …, B. K. Kobilka. 2011. Structure of a nanobody-stabilized active state of the β2 adrenoceptor. *Nature.* 469:175–180.
19. Tejero, O., F. Pamula, …, C. J. Tsai. 2024. Active state structures of a bistable visual opsin bound to G proteins. *Nat. Commun.* 15:8928.
20. Palczewski, K., T. Kumasaka, …, M. Miyano. 2000. Crystal structure of rhodopsin: AG protein-coupled receptor. *Science.* 289:739–745.
21. Hubbard, R., and R. C. S. George. 1958. The rhodopsin system of the squid. *J. Gen. Physiol.* 41:501–528.
22. Tsukamoto, H., and A. Terakita. 2010. Diversity and functional properties of bistable pigments. *Photochem. Photobiol. Sci.* 9:1435–1443.
23. Koyanagi, M., and A. Terakita. 2014. Diversity of animal opsin-based pigments and their optogenetic potential. *Biochim. Biophys. Acta.* 1837:710–716.
24. Nagata, T., M. Koyanagi, …, A. Terakita. 2012. Depth perception from image defocus in a jumping spider. *Science.* 335:469–471.
25. Varma, N., E. Mutt, …, E. Lesca. 2019. Crystal structure of jumping spider rhodopsin-1 as a light sensitive GPCR. *Proc. Natl. Acad. Sci. USA.* 116:14547–14556.
26. Bernetti, M., S. Bosio, …, M. Masetti. 2024. Probing allosteric communication with combined molecular dynamics simulations and network analysis. *Curr. Opin. Struct. Biol.* 86:102820.
27. Telari, E., A. Tinti, …, A. Giacomello. 2023. Charting nanocluster structures via convolutional neural networks. *ACS Nano.* 17:21287–21296.
28. Guex, N., M. C. Peitsch, and T. Schwede. 2009. Automated comparative protein structure modeling with SWISS-MODEL and Swiss-PdbViewer: A historical perspective. *Electrophoresis.* 30:S162–S173.
29. Jurrus, E., D. Engel, …, N. A. Baker. 2018. Improvements to the APBS biomolecular solvation software suite. *Protein Sci.* 27:112–128.
30. Church, J. R., J. M. H. Olsen, and I. Schapiro. 2021. The impact of retinal configuration on the protein–chromophore interactions in bistable jumping spider rhodopsin-1. *Molecules.* 27:71.
31. Jo, S., T. Kim, …, W. Im. 2008. CHARMM-GUI: a web-based graphical user interface for CHARMM. *J. Comput. Chem.* 29:1859–1865.
32. Wu, E. L., X. Cheng, …, W. Im. 2014. CHARMM-GUI membrane builder toward realistic biological membrane simulations. *J. Comput. Chem.* 35:1997–2004.
33. Mark, P., and L. Nilsson. 2001. Structure and dynamics of the TIP3P, SPC, and SPC/E water models at 298 K. *J. Phys. Chem. A.* 105:9954–9960.
34. Abraham, M. J., T. Murtola, …, E. Lindahl. 2015. GROMACS: High performance molecular simulations through multi-level parallelism from laptops to supercomputers. *SoftwareX.* 1–2:19–25.
35. Maier, J. A., C. Martinez, …, C. Simmerling. 2015. ff14SB: improving the accuracy of protein side chain and backbone parameters from ff99SB. *J. Chem. Theor. Comput.* 11:3696–3713.
36. Dickson, C. J., B. D. Madej, …, R. C. Walker. 2014. Lipid14: the amber lipid force field. *J. Chem. Theor. Comput.* 10:865–879.
37. Martyna, G. J., D. J. Tobias, and M. L. Klein. 1994. Constant pressure molecular dynamics algorithms. *J. Chem. Phys.* 101:4177–4189.
38. Feller, S. E., Y. Zhang, …, B. R. Brooks. 1995. Constant pressure molecular dynamics simulation: The Langevin piston method. *J. Chem. Phys.* 103:4613–4621.

39. Darden, T., D. York, and L. Pedersen. 1993. Particle mesh Ewald: An N·logNmethod for Ewald sums in large systems. *J. Chem. Phys.* 98:10089–10092.
40. Humphrey, W., A. Dalke, and K. Schulten. 1996. VMD: visual molecular dynamics. *J. Mol. Graph.* 14:33-8–27-8.
41. Yao, X.-Q., M. Momin, and D. Hamelberg. 2019. Establishing a Framework of Using Residue–Residue Interactions in Protein Difference Network Analysis. *J. Chem. Inf. Model.* 59:3222–3228.
42. Westerlund, A. M., O. Fleetwood, …, L. Delemotte. 2020. Network analysis reveals how lipids and other cofactors influence membrane protein allostery. *J. Chem. Phys.* 153:141103.
43. Bassetto, C. A. Z., Jr., F. Costa, …, A. Giacomello. 2023. Noncanonical electromechanical coupling paths in cardiac hERG potassium channel. *Nat. Commun.* 14:1110.
44. Costa, F., R. Ocello, …, M. Masetti. 2023. Integrated Approach Including Docking, MD Simulations, and Network Analysis Highlights the Action Mechanism of the Cardiac hERG Activator RPR260243. *J. Chem. Inf. Model.* 63:4888–4899.
45. Dijkstra, E. W. 1959. A note on two problems in connexion with graphs. *Numer. Math.* 1:269–271.
46. Telari, E., A. Tinti, …, A. Giacomello. 2025. Inherent structural descriptors via machine learning. *Rep. Prog. Phys.* 88:6.
47. Hinton, G. E., and R. R. Salakhutdinov. 2006. Reducing the dimensionality of data with neural networks. *Science.* 313:504–507.
48. Paszke, A., S. Gross, …, S. Chintala. 2019. Pytorch: an imperative style, high-performance deep learning library. *Adv. Neural Inf. Process Syst.* 32
49. LeCun, Y., L. Bottou, …, P. Haffner. 1998. Gradient-based learning applied to document recognition. *Proc. IEEE.* 86:2278–2324.
50. LeCun, Y., Y. Bengio, and G. Hinton. 2015. Deep learning. *Nature.* 521:436–444.
51. Kingma, D. P. 2014. Adam: A method for stochastic optimization. Preprint at arXiv. https://doi.org/10.48550/arXiv:1412.6980.
52. Pedregosa, F., G. Varoquaux, …, E. Duchesnay. 2011. Scikit-learn: Machine Learning in Python. *J. Mach. Learn. Res.* 12:2825–2830.
53. Ward, J. H., Jr. 1963. Hierarchical grouping to optimize an objective function. *J. Am. Stat. Assoc.* 58:236–244.
54. Bertalan, É., E. Lesca, …, A.-N. Bondar. 2021. C-Graphs tool with graphical user interface to dissect conserved hydrogen-bond networks: Applications to visual rhodopsins. *J. Chem. Inf. Model.* 61:5692–5707.
55. Zhou, X. E., K. Melcher, and H. E. Xu. 2012. Structure and activation of rhodopsin. *Acta Pharmacol. Sin.* 33:291–299.
56. Kimata, N., A. Pope, …, S. O. Smith. 2016. Free backbone carbonyls mediate rhodopsin activation. *Nat. Struct. Mol. Biol.* 23:738–743.
57. Han, M., S. W. Lin, …, T. P. Sakmar. 1996. Functional interaction of transmembrane helices 3 and 6 in rhodopsin: replacement of phenylalanine 261 by alanine causes reversion of phenotype of a glycine 121 replacement mutant. *J. Biol. Chem.* 271:32337–32342.
58. Han, M., S. O. Smith, and T. P. Sakmar. 1998. Constitutive activation of opsin by mutation of methionine 257 on transmembrane helix 6. *Biochemistry.* 37:8253–8261.
59. Madabushi, S., A. K. Gross, …, O. Lichtarge. 2004. Evolutionary trace of G protein-coupled receptors reveals clusters of residues that determine global and class-specific functions. *J. Biol. Chem.* 279:8126–8132.
60. Elgeti, M., R. Kazmin, …, F. J. Bartl. 2011. Conserved Tyr2235. 58 plays different roles in the activation and G-protein interaction of rhodopsin. *J. Am. Chem. Soc.* 133:7159–7165.
61. Ren, Z., P. X. Ren, …, X. Yang. 2016. Transmembrane helices tilt, bend, slide, torque, and unwind between functional states of rhodopsin. *Sci. Rep.* 6:34129.
62. Li, J., P. C. Edwards, …, G. F. X. Schertler. 2004. Structure of bovine rhodopsin in a trigonal crystal form. *J. Mol. Biol.* 343:1409–1438.
63. Singhal, A., Y. Guo, …, J. Standfuss. 2016. Structural role of the T94I rhodopsin mutation in congenital stationary night blindness. *EMBO Rep.* 17:1431–1440.

Biophysical Journal, Volume *125*

Supplemental information

Mapping signal transduction in bistable jumping spider rhodopsin 1

Flavio Costa, Emanuele Telari, Daniel Moreno-Rodríguez, Simone Meloni, Jógvan Magnus Haugaard Olsen, Alberto Giacomello, and Giovanni Di Muccio

Supporting Information: Mapping Signal Transduction in Bistable Jumping Spider Rhodopsin 1

Flavio Costa,† Emanuele Telari,‡ Daniel Moreno-Rodríguez,† Simone Meloni,¶ Jógvan Magnus Haugaard Olsen,§ Alberto Giacomello,† and Giovanni Di Muccio*,†,‖

†*Dipartimento di Ingegneria Meccanica e Aerospaziale, Sapienza Università di Roma, Via Eudossiana 18, 00184, Rome, Italy*

‡*Departament de Ciència de Materials i Química Física & Institut de Quimica Teòrica i Computacional (IQTCUB), Universitat de Barcelona, c/Martí i Franquès 1, 08028 Barcelona, Spain*

¶*Department of Chemical, Pharmaceutical and Agricultural Sciences, University of Ferrara, Ferrara, 44121 Italy*

§*DTU Chemistry, Technical University of Denmark, DK-2800 Kongens Lyngby, Denmark*

‖*NY-Masbic, Department of Life and Environmental Sciences, Marche Polytechnic University, Via Brecce Bianche, 60131, Ancona, Italy*

E-mail: g.dimuccio@univpm.it

```
----------------------------------------------------------------
        Layer (type)               Output Shape         Param #
================================================================
            Conv2d-1        [-1, 16, 130, 130]             160
         MaxPool2d-2          [-1, 16, 65, 65]               0
              ReLU-3          [-1, 16, 65, 65]               0
       BatchNorm2d-4          [-1, 16, 65, 65]              32
            Conv2d-5          [-1, 32, 65, 65]           4,640
         MaxPool2d-6          [-1, 32, 32, 32]               0
              ReLU-7          [-1, 32, 32, 32]               0
       BatchNorm2d-8          [-1, 32, 32, 32]              64
            Conv2d-9          [-1, 64, 32, 32]          18,496
        MaxPool2d-10          [-1, 64, 16, 16]               0
             ReLU-11          [-1, 64, 16, 16]               0
      BatchNorm2d-12          [-1, 64, 16, 16]             128
           Conv2d-13          [-1, 64, 16, 16]          36,928
        MaxPool2d-14            [-1, 64, 8, 8]               0
             ReLU-15            [-1, 64, 8, 8]               0
      BatchNorm2d-16            [-1, 64, 8, 8]             128
           Conv2d-17            [-1, 64, 8, 8]          36,928
        MaxPool2d-18            [-1, 64, 4, 4]               0
             ReLU-19            [-1, 64, 4, 4]               0
      BatchNorm2d-20            [-1, 64, 4, 4]             128
           Conv2d-21           [-1, 128, 4, 4]          73,856
        MaxPool2d-22           [-1, 128, 2, 2]               0
             ReLU-23           [-1, 128, 2, 2]               0
      BatchNorm2d-24           [-1, 128, 2, 2]             256
          Flatten-25                  [-1, 512]               0
           Linear-26                    [-1, 2]           1,026
           Linear-27                  [-1, 512]           1,536
             ReLU-28                  [-1, 512]               0
         Upsample-29           [-1, 128, 4, 4]               0
  ConvTranspose2d-30            [-1, 64, 4, 4]          73,792
             ReLU-31            [-1, 64, 4, 4]               0
      BatchNorm2d-32            [-1, 64, 4, 4]             128
         Upsample-33            [-1, 64, 8, 8]               0
  ConvTranspose2d-34            [-1, 64, 8, 8]          36,928
             ReLU-35            [-1, 64, 8, 8]               0
      BatchNorm2d-36            [-1, 64, 8, 8]             128
         Upsample-37          [-1, 64, 16, 16]               0
  ConvTranspose2d-38          [-1, 64, 16, 16]          36,928
             ReLU-39          [-1, 64, 16, 16]               0
      BatchNorm2d-40          [-1, 64, 16, 16]             128
         Upsample-41          [-1, 64, 32, 32]               0
  ConvTranspose2d-42          [-1, 32, 32, 32]          18,464
             ReLU-43          [-1, 32, 32, 32]               0
      BatchNorm2d-44          [-1, 32, 32, 32]              64
         Upsample-45          [-1, 32, 64, 64]               0
  ConvTranspose2d-46          [-1, 16, 64, 64]           4,624
             ReLU-47          [-1, 16, 64, 64]               0
      BatchNorm2d-48          [-1, 16, 64, 64]              32
         Upsample-49        [-1, 16, 128, 128]               0
           Conv2d-50         [-1, 1, 130, 130]             145
================================================================
Total params: 345,667
Trainable params: 345,667
Non-trainable params: 0
----------------------------------------------------------------
Input size (MB): 0.06
Forward/backward pass size (MB): 13.12
Params size (MB): 1.32
Estimated Total Size (MB): 14.51
----------------------------------------------------------------
```

Figure S1: Network configuration as printed by torchsummary. The matrices fed to the network were padded with a row and column of zeros to reach a size of 130×130, from the initial size of 129×129, being this one more complicated to handle with the max poolings and upscalings

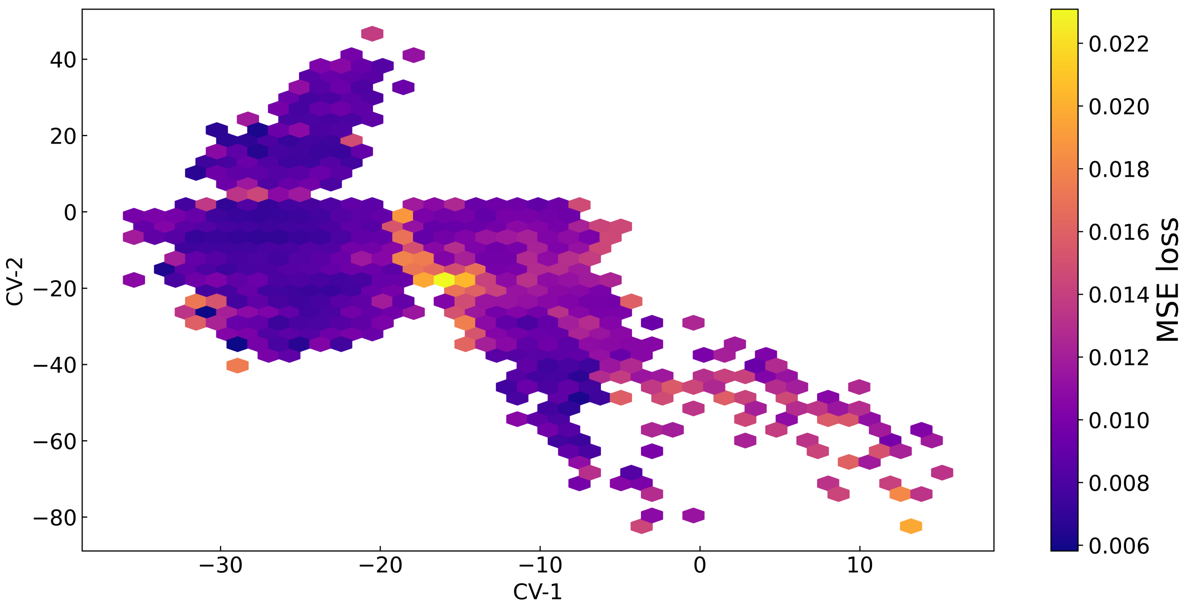

Figure S2: Heat map of the MSE loss values across the reduced space.

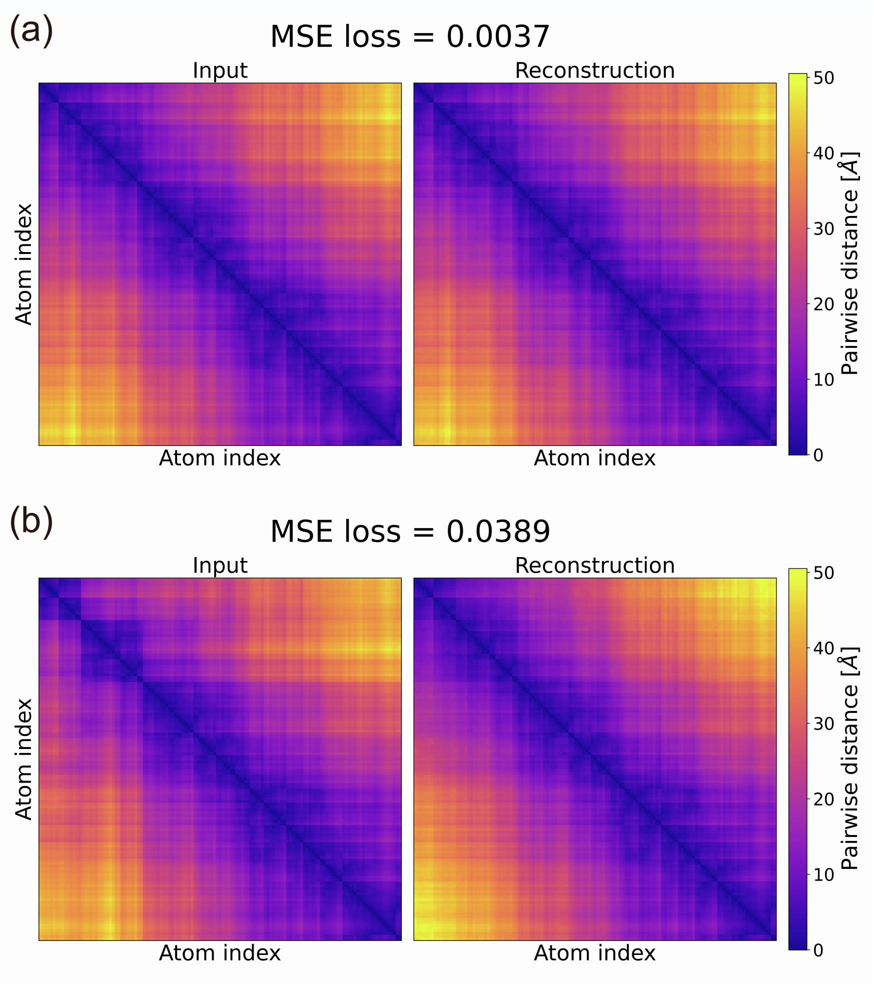

Figure S3: (a) Comparison between input (left) and reconstructed output (right) of the network for the sample associated to the best (lowest) MSE. The two heat maps share the same color scale reported by the colorbar on the right. (b) Comparison between input and output for the sample showing the highest MSE value. The two heat maps share the same color scale reported by the colorbar on the right.

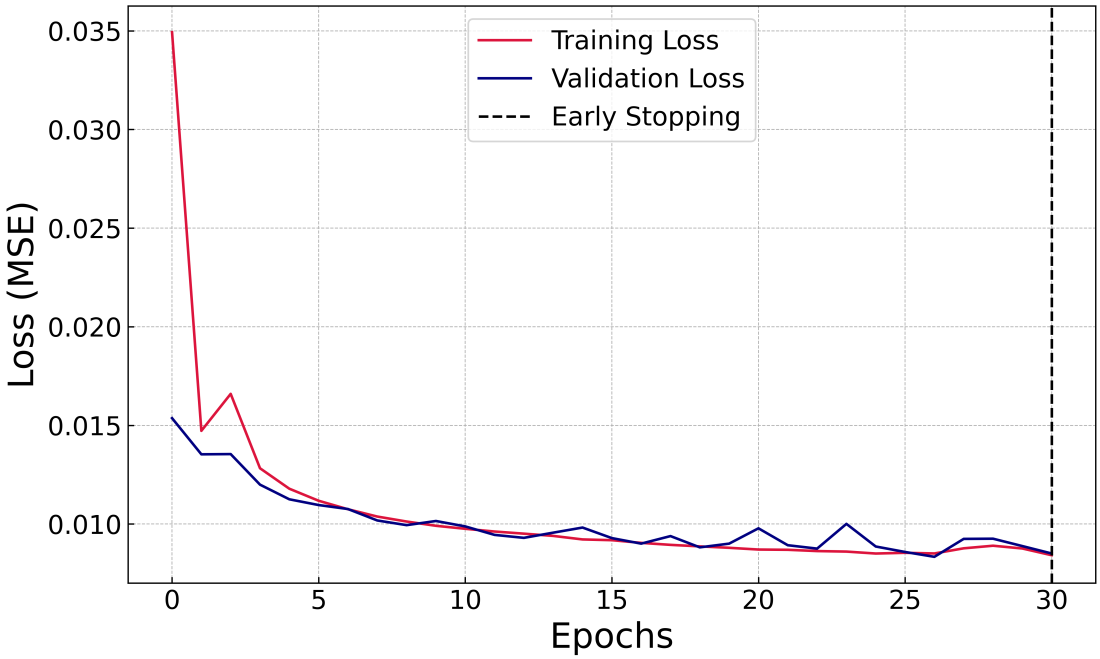

Figure S4: Plots of the training and validation loss during the training. The early stopping stopped the training after 30 epochs and the best model saved was the one of epoch 30.

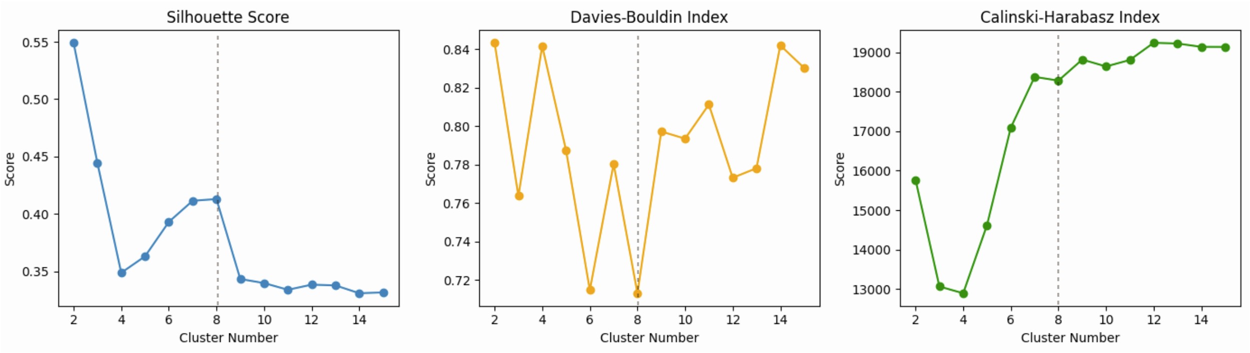

Figure S5: Plot of three metrics used to evaluate the clustering performance and the optimal number of clusters. Dotted lines indicate the final number of clusters used.

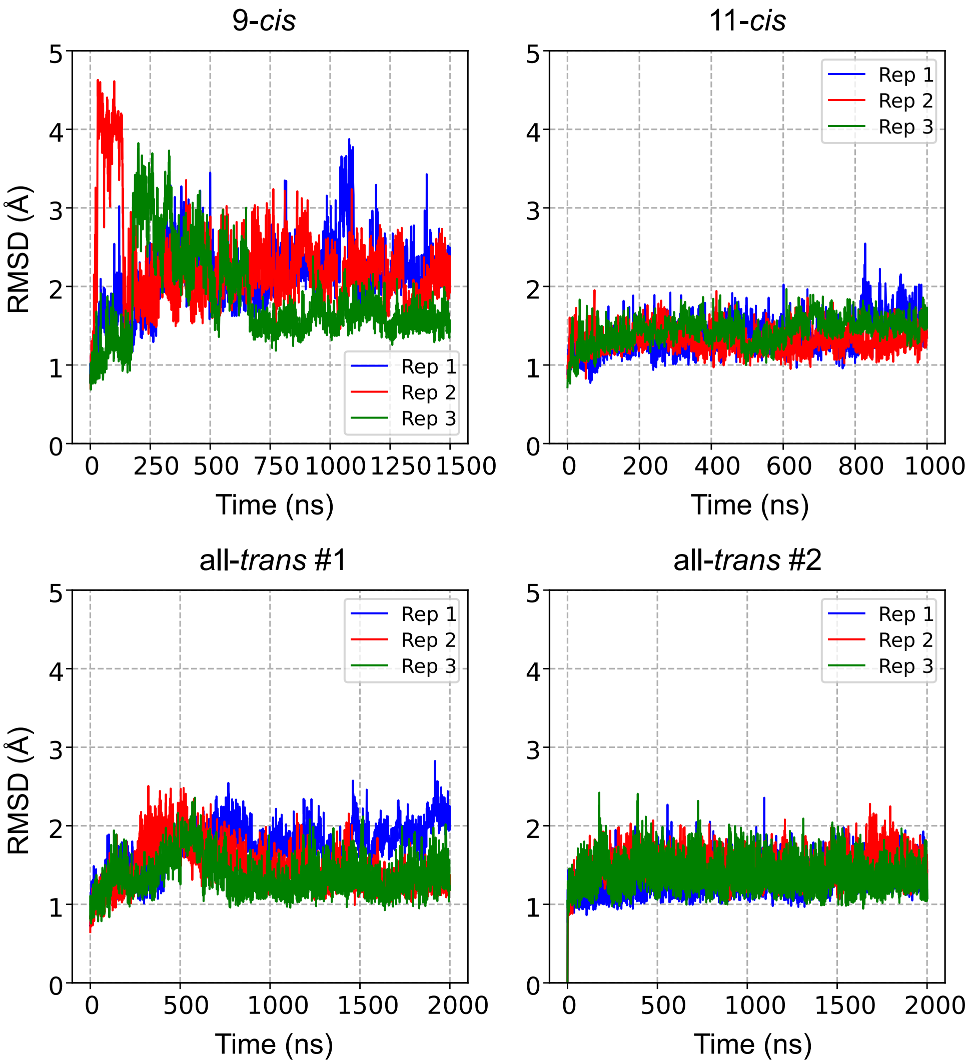

Figure S6: Root Mean Square Displacement calculations of each replica where only the Cα were considered and the reference conformation corresponded to that at the beginning of the dynamics.

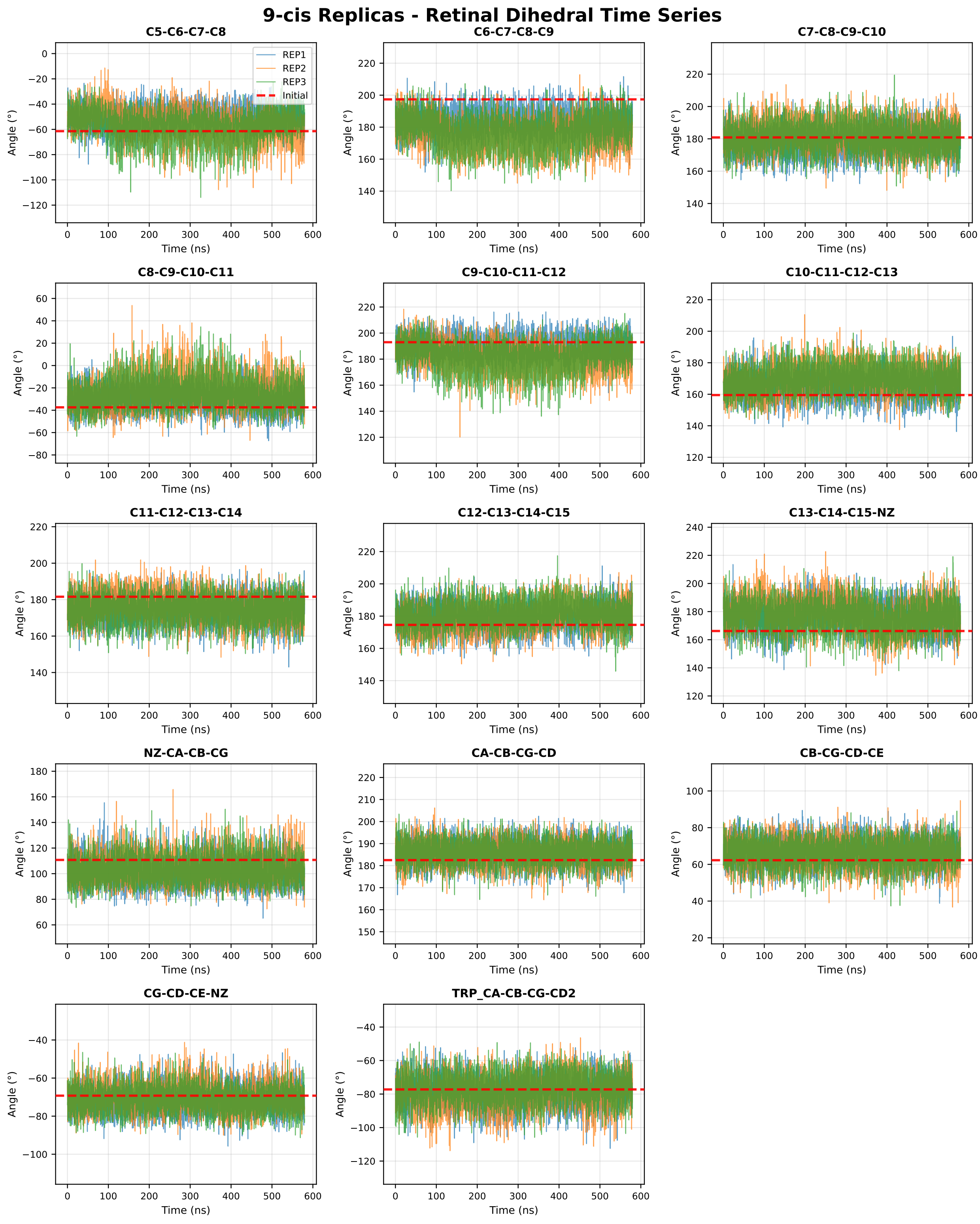

Figure S7: MD retinal dihedral angles for **9-cis** isomer conformation. Starting structure angle is reported in dashed red line. Summary Table is reported in Table S1.

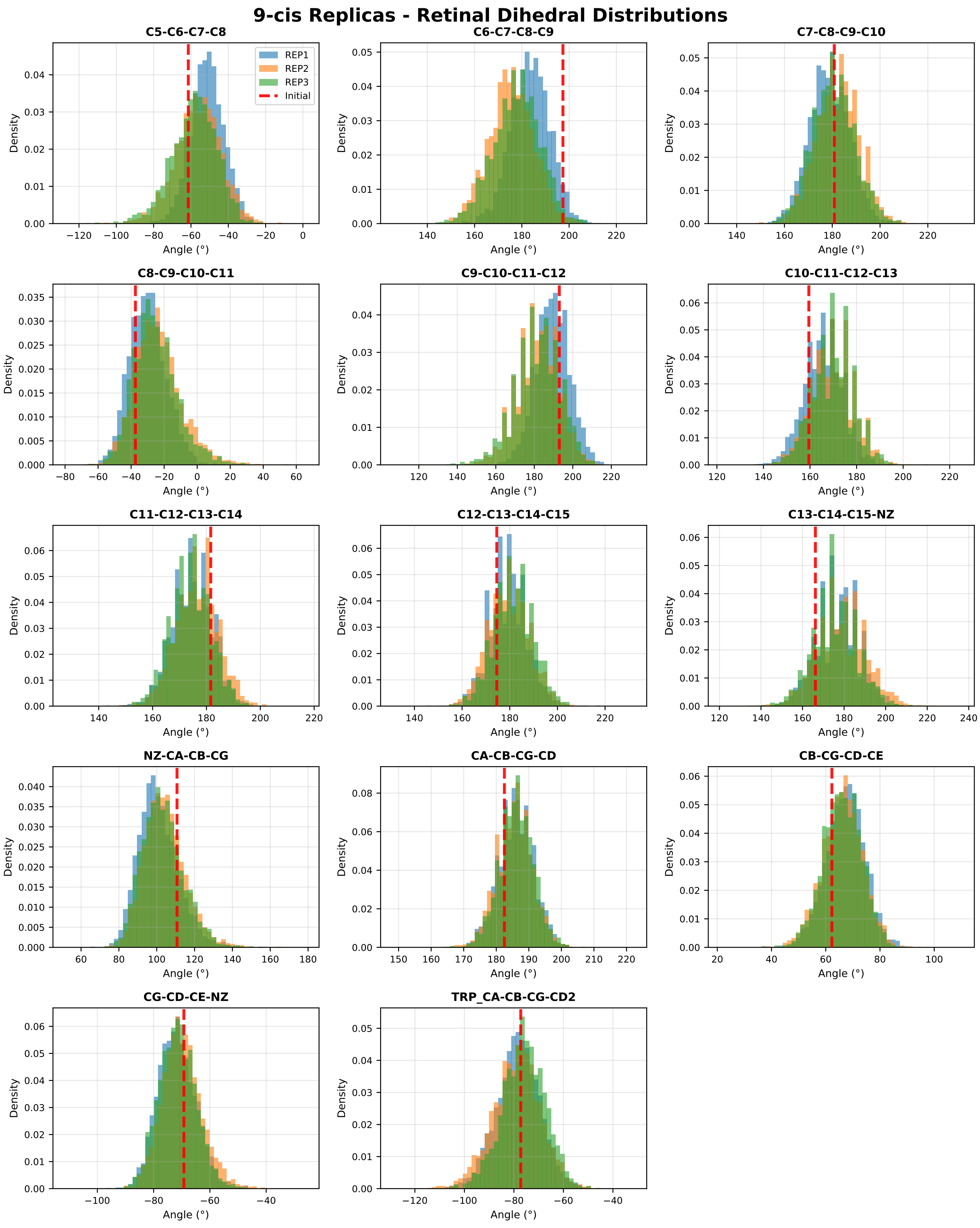

Figure S8: MD retinal dihedral angles for **9-cis** isomer conformation. Starting structure angle is reported in dashed red line. Summary Table is reported in Table S1.

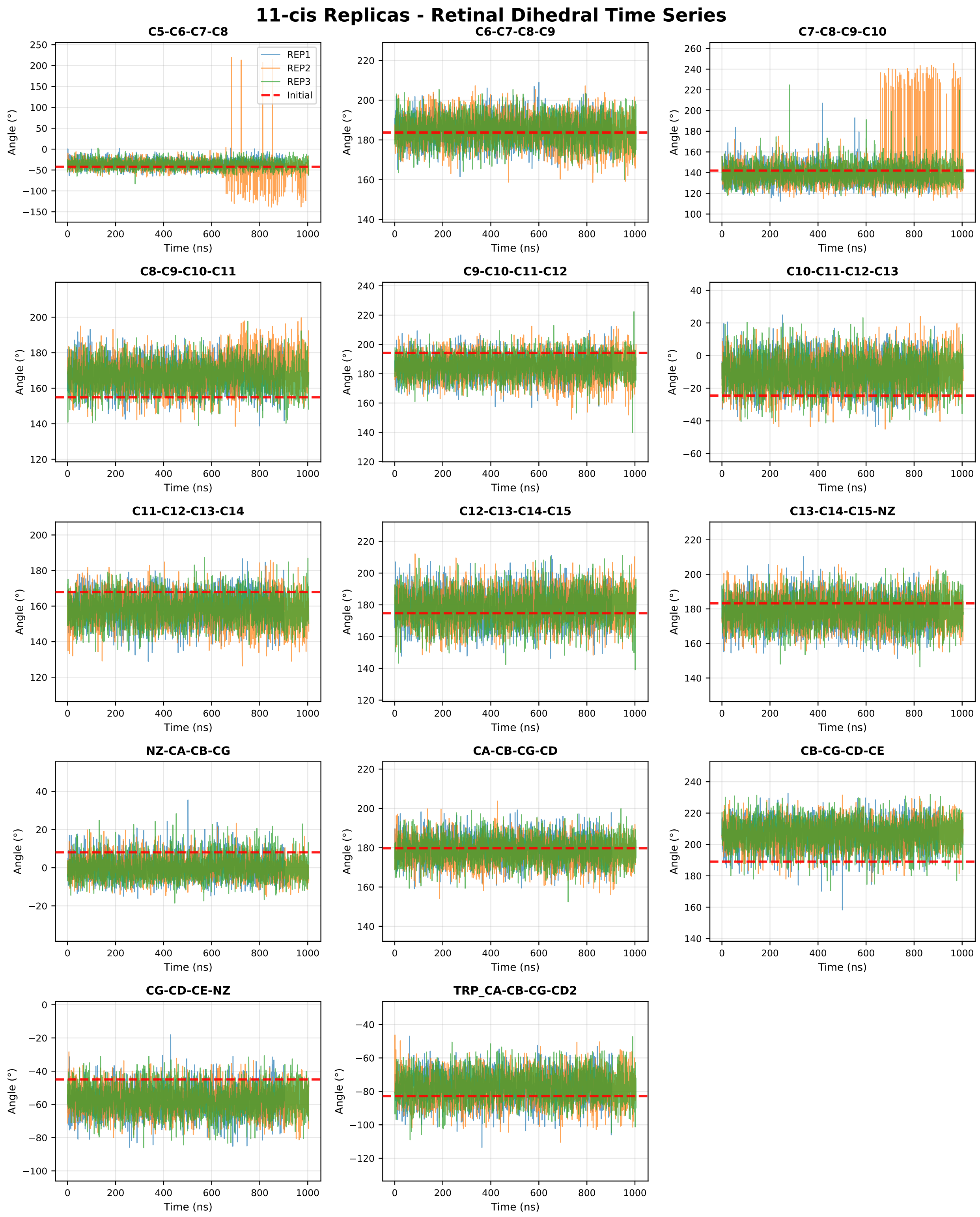

Figure S9: MD retinal dihedral angles for **11-cis** isomer conformation. Starting structure angle is reported in dashed red line. Summary Table is reported in Table S2.

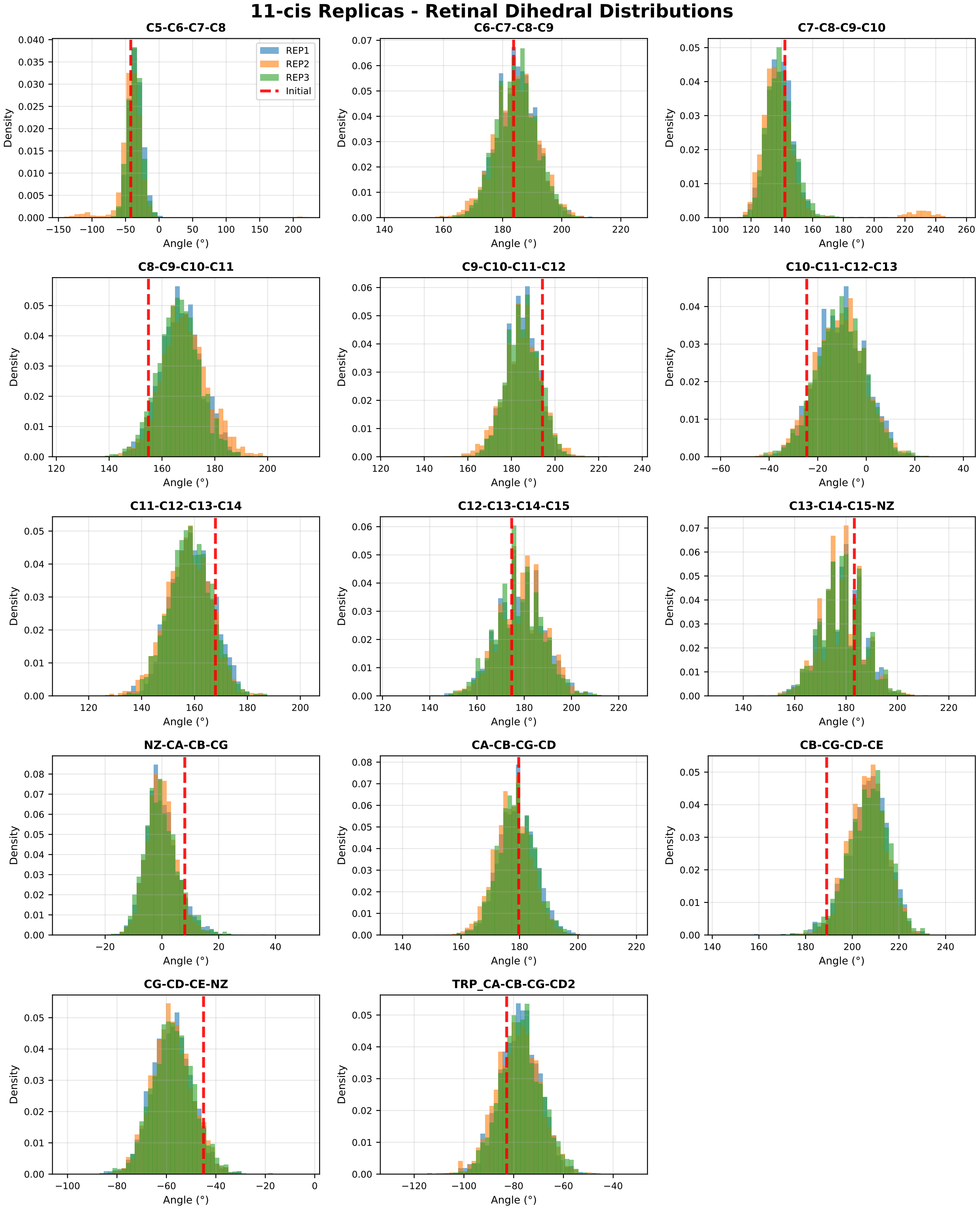

Figure S10: MD retinal dihedral angles for **11-cis** isomer conformation. Starting structure angle is reported in dashed red line. Summary Table is reported in Table S2.

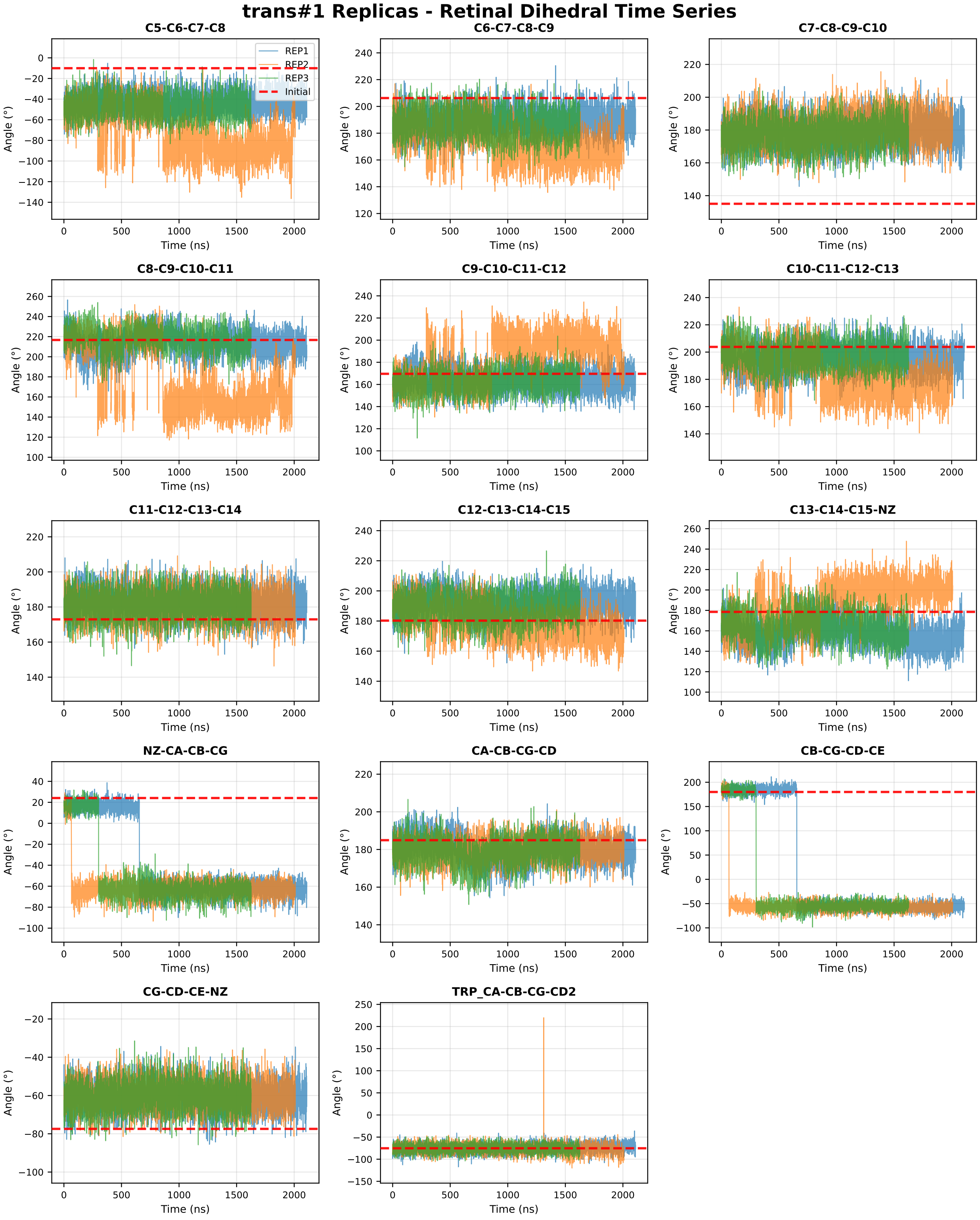

Figure S11: MD retinal dihedral angles for **all-*trans*#1** isomer conformation. Starting structure angle is reported in dashed red line. Summary Table is reported in Table S3.

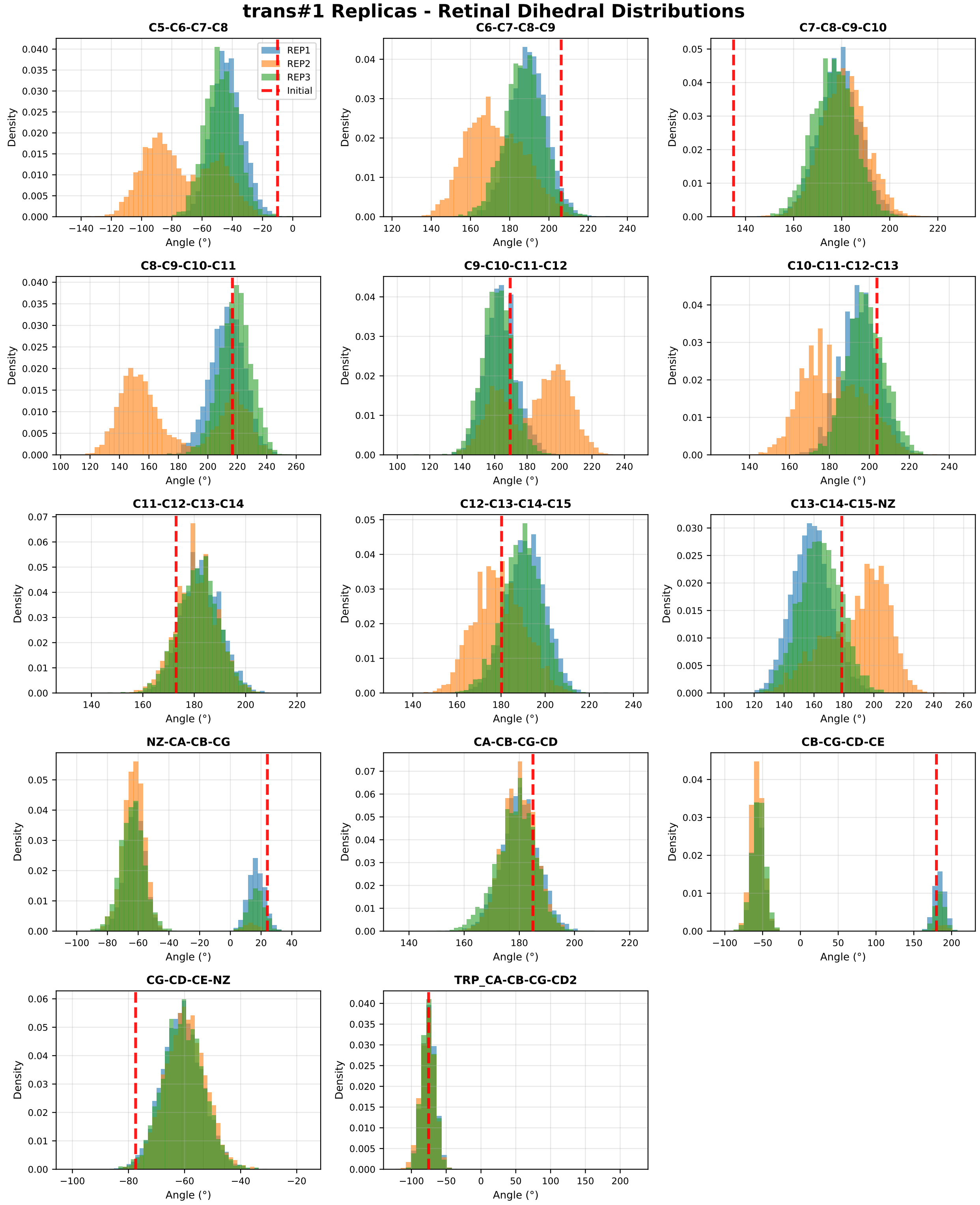

Figure S12: MD retinal dihedral angles for **all-*trans*#1** isomer conformation. Starting structure angle is reported in dashed red line. Summary Table is reported in Table S3.

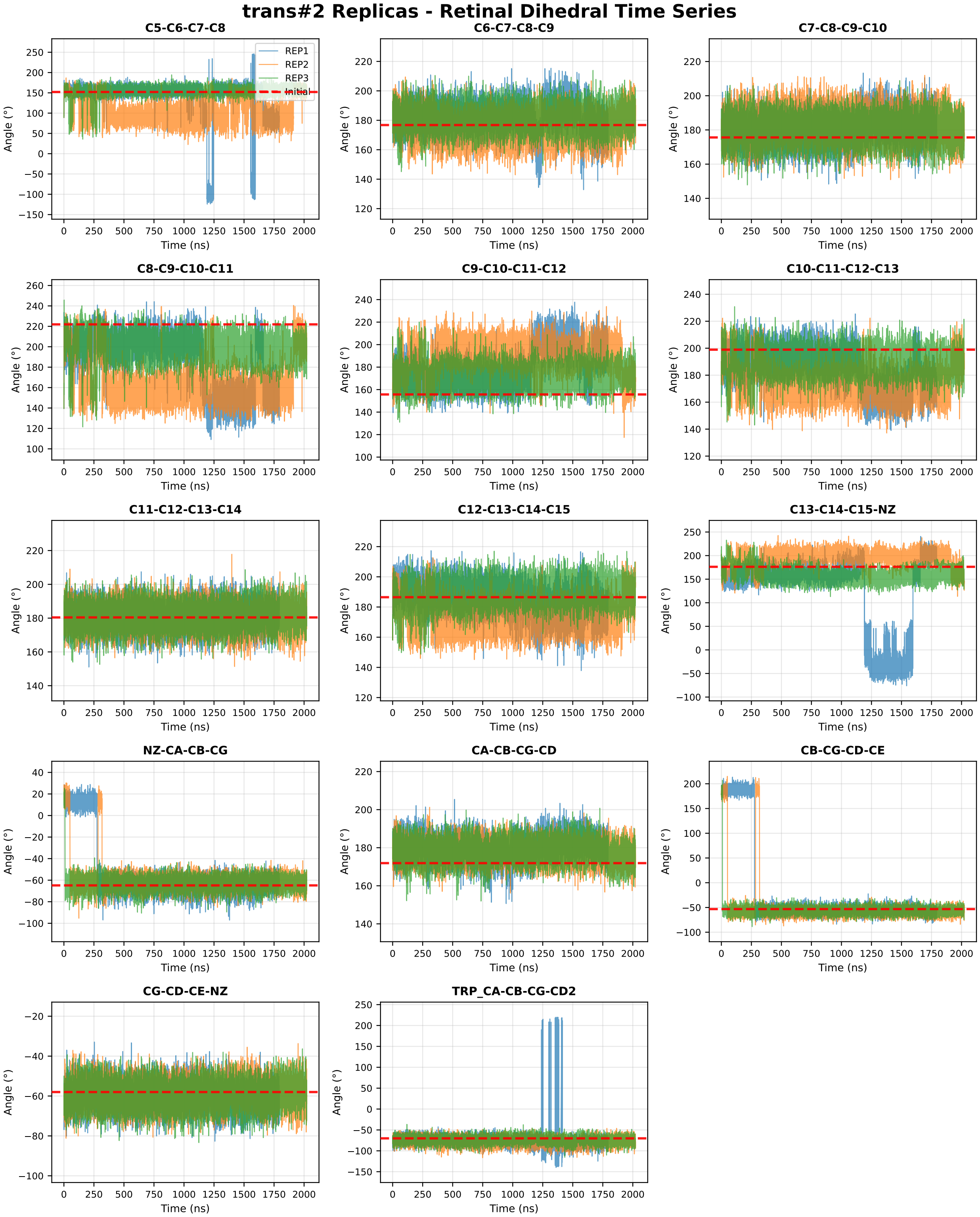

Figure S13: MD retinal dihedral angles for an alternative **all-*trans*#2** isomer conformation. Starting structure angle is reported in dashed red line. Summary Table is reported in Table S4.

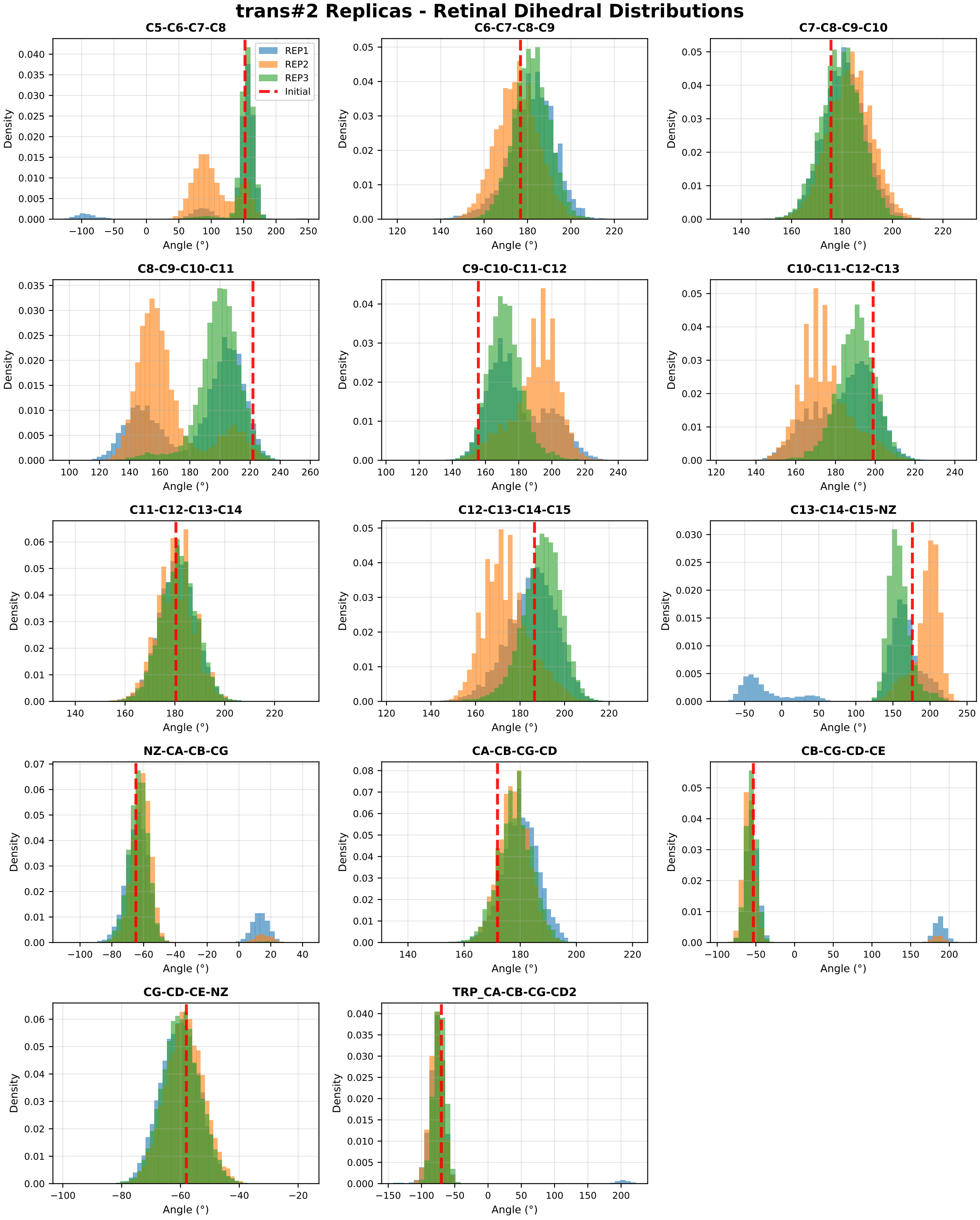

Figure S14: MD retinal dihedral angles for an alternative **all-*trans*#2** isomer conformation. Starting structure angle is reported in dashed red line. Summary Table is reported in Table S4.

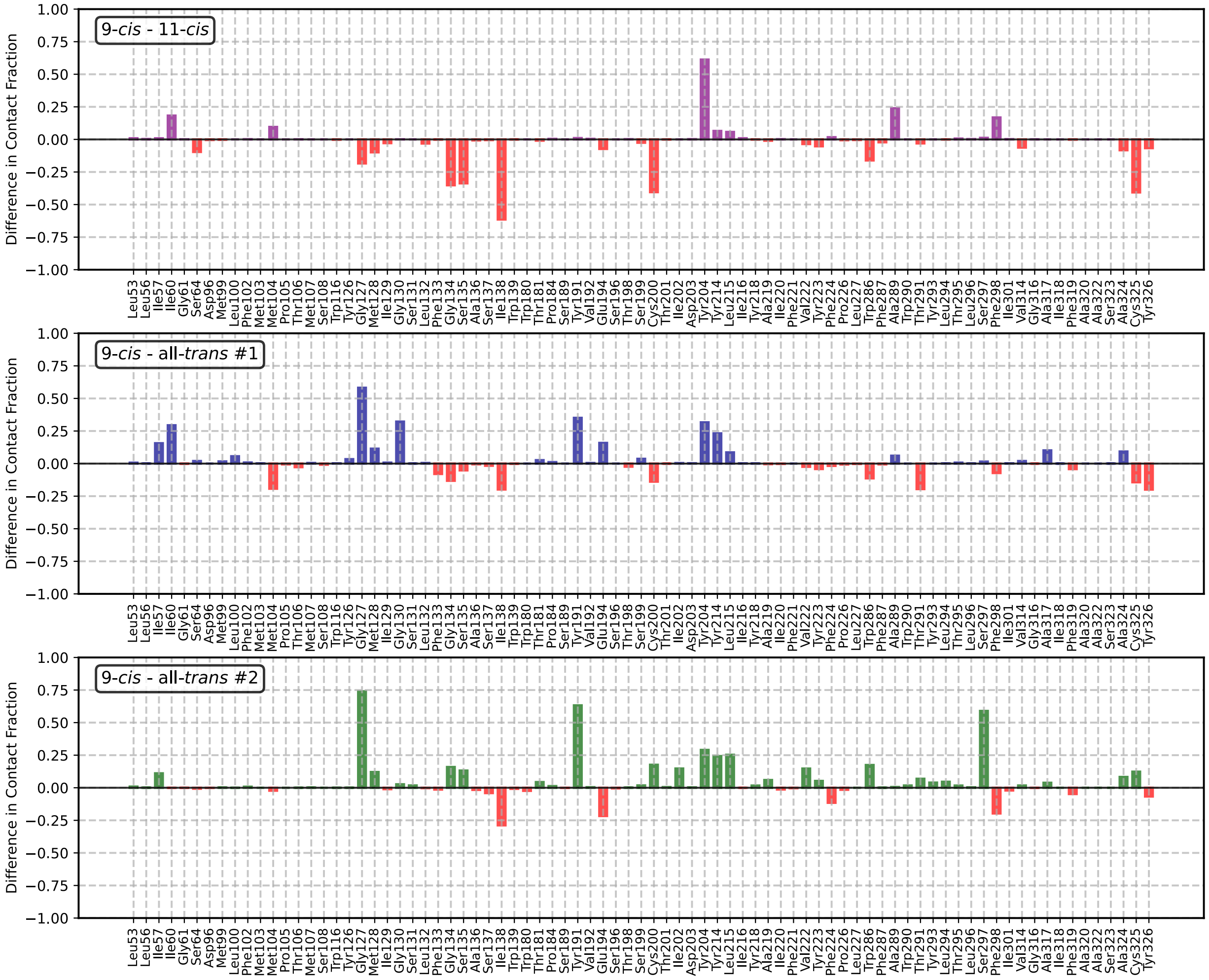

Figure S15: Differences in contact fraction between 9-*cis* and 11-*cis*, all-*trans* #1, and all-*trans* #2 systems. A difference in contact fraction equal to 1 indicates that the interaction persists during the whole trajectory in the 9-*cis* while equal to -1 in the others.

```
Bovine   55 NFLTLYVTVQHKKLRTPLNYILLNLAVADLFMVFGGFTTTLYTSLHGYFVFGPTGCNLEG
JSR1     68 NGVVMYLMMTVKNLRTPGNFLVLNLALSDFGMLFFMMPTMSINCFAETWVIGPFMCELYG
            *    *     * **** *   ****  *  * *    *          * **  * * *

Bovine  115 FFATLGGEIALWSLVVLAIERYVVVCKPMSNFRFGENHAIMGVAFTWVMALACAAPPLVG
JSR1    128 MIGSLFGSASIWSLVMITLDRYNVIVKGMAGKPLTKVGALLRMLFVWIWSLGWTIAPMYG
                * *    ****     ** *  * *         *     * *   *     *  *

Bovine  175 WSRYIPEGMQCSCGIDYYTPHEETNNESFVIYMFVVHFIIPLIVIFFCYGQLVFTVKEAA
JSR1    188 WSRYVPEGSMTSCTIDYIDT--AINPMSYLIAYAIFVYFVPLFIIIYCYAFIVMQVAAHE
            **** ***   ** ***       *  *  *         **  *  **   *  *

Bovine  235 AQQQESATTQK--------------AEKEVTRMVIIMVIAFLICWLPYAGVAFYIFTHQG
JSR1    246 KSLREQAKKMNIKSLRSNEDNKKASAEFRLAKVAFMTICCWFMAWTPYLTLSFLGIFSDR
                * *                  **                 * **    *

Bovine  281 SDFGPIFMTIPAFFAKTSAVYNPVIYIMMNKQFRNCM---VTTLCCGKNPLGDDEASTTV
JSR1    306 TWLTPMTSVWGAIFAKASACYNPIVYGISHPKYRAALHDKFPCLKCGSDSPKGDSASTVA
                *      * *** ** ***  *       *         * **      * ***

Bovine  338 SKTETSQ
JSR1    366 ESEKAGE
```

Figure S16: Sequence alignment of Bovine Rhodopsin (UniProt ID P02699) and Jumping Spider Rhodopsin 1 (UniProt ID B1B1U5).

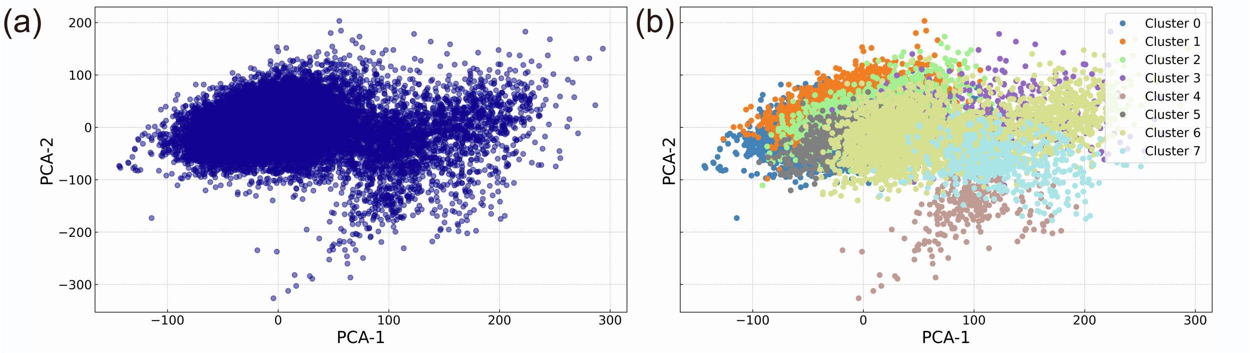

Figure S17: (a) PCA space obtained using the same data for the autoencoder training. (b) Points are colored according to the clustering performed in the autoencoder space of Figure 5 in the main text.

Table S1: Comparison of retinal dihedral angles for **9-cis** isomer: Molecular dynamics (MD) averages versus starting structure PDB 6I9K. Angles are reported in degrees (°). Key dihedrals indicating cis configurations (ϕ_4 and ϕ_6) are highlighted. SD is the standard deviation of the concatenated dataset.

**Dihedral Label**	**Atoms Involved**	**Description**	**6I9K (°)**	**MD Avg. ± SD (°)**	**Δ (°)**
ϕ_1	C5-C6-C7-C8	6-7 bond torsion	-61.4	-55.3 ± 12.1	6.1
ϕ_2	C6-C7-C8-C9	7-8 bond torsion	197.4	178.9 ± 10.0	-18.5
ϕ_3	C7-C8-C9-C10	8-9 bond torsion	180.9	180.6 ± 9.0	-0.3
ϕ_4	C8-C9-C10-C11	**9-10 bond**	-37.4	-26.2 ± 13.7	11.2
ϕ_5	C9-C10-C11-C12	10-11 bond torsion	193.0	184.4 ± 11.3	-8.6
ϕ_6	C10-C11-C12-C13	**11-12 bond**	159.5	168.4 ± 8.8	8.9
ϕ_7	C11-C12-C13-C14	12-13 bond torsion	181.6	175.1 ± 7.6	-6.5
ϕ_8	C12-C13-C14-C15	13-14 bond torsion	174.6	180.1 ± 8.3	5.5
ϕ_9	C13-C14-C15-N	14-15-Schiff base torsion	45.8	59.3 ± 16.1	13.5
χ_1	N-CA-CB-CG	Lysine sidechain torsion 1	96.6	79.6 ± 9.1	-17.0
χ_2	CA-CB-CG-CD	Lysine sidechain torsion 2	182.5	185.9 ± 5.3	3.4
χ_3	CB-CG-CD-CE	Lysine sidechain torsion 3	62.3	66.5 ± 7.2	4.2
χ_4	CG-CD-CE-NZ	Lysine sidechain torsion 4	-69.2	-71.1 ± 6.8	-1.8

Table S2: Comparison of retinal dihedral angles for the **11-*cis*** isomer: Molecular dynamics (MD) averages versus starting structure. Angles are reported in degrees (°). The key dihedral indicating the 11-*cis* configuration (ϕ_6) is highlighted.

**Dihedral Label**	**Atoms Involved**	**Description**	**Start. (°)**	**MD Avg. ± SD (°)**	**Δ (°)**
ϕ_1	C5-C6-C7-C8	6-7 bond torsion	-42.16	-33.67 ± 32.85	8.49
ϕ_2	C6-C7-C8-C9	7-8 bond torsion	183.73	184.66 ± 6.89	0.93
ϕ_3	C7-C8-C9-C10	8-9 bond torsion	141.99	139.47 ± 13.89	-2.52
ϕ_4	C8-C9-C10-C11	**9-10 bond**	154.88	167.35 ± 8.30	12.46
ϕ_5	C9-C10-C11-C12	10-11 bond torsion	194.23	185.00 ± 7.97	-9.22
ϕ_6	C10-C11-C12-C13	**11-12 bond**	-24.50	-10.55 ± 10.12	13.95
ϕ_7	C11-C12-C13-C14	12-13 bond torsion	167.91	158.26 ± 8.17	-9.65
ϕ_8	C12-C13-C14-C15	13-14 bond torsion	174.69	178.38 ± 9.96	3.68
ϕ_9	C13-C14-C15-N	14-15-Schiff base torsion	183.23	178.45 ± 8.36	-4.77
χ_1	N-CA-CB-CG	Lysine sidechain torsion 1	8.05	-0.12 ± 5.72	-8.18
χ_2	CA-CB-CG-CD	Lysine sidechain torsion 2	179.71	178.58 ± 6.32	-1.13
χ_3	CB-CG-CD-CE	Lysine sidechain torsion 3	189.04	206.63 ± 8.54	17.58
χ_4	CG-CD-CE-NZ	Lysine sidechain torsion 4	-44.97	-57.61 ± 8.25	-12.64

Table S3: Comparison of retinal dihedral angles for the **all-*trans*#1** isomer: Molecular dynamics (MD) averages versus starting structure. Angles are reported in degrees (°).

**Dihedral Label**	**Atoms Involved**	**Description**	**Start. (°)**	**MD Avg. ± SD (°)**	**Δ (°)**
ϕ_1	C5-C6-C7-C8	6-7 bond torsion	-10.03	-54.73 ± 20.81	-44.70
ϕ_2	C6-C7-C8-C9	7-8 bond torsion	206.24	183.14 ± 13.75	-23.09
ϕ_3	C7-C8-C9-C10	8-9 bond torsion	135.03	178.84 ± 9.00	43.80
ϕ_4	C8-C9-C10-C11	**9-10 bond**	216.68	203.04 ± 28.45	-13.64
ϕ_5	C9-C10-C11-C12	10-11 bond torsion	169.59	169.83 ± 17.50	0.23
ϕ_6	C10-C11-C12-C13	**11-12 bond**	203.80	191.91 ± 13.76	-11.88
ϕ_7	C11-C12-C13-C14	12-13 bond torsion	172.94	181.61 ± 7.83	8.66
ϕ_8	C12-C13-C14-C15	13-14 bond torsion	180.25	186.55 ± 11.13	6.29
ϕ_9	C13-C14-C15-N	14-15-Schiff base torsion	178.53	170.34 ± 21.15	-8.19
χ_1	N-CA-CB-CG	Lysine sidechain torsion 1	24.11	-49.12 ± 31.28	-73.23
χ_2	CA-CB-CG-CD	Lysine sidechain torsion 2	184.93	179.56 ± 6.66	-5.36
χ_3	CB-CG-CD-CE	Lysine sidechain torsion 3	179.95	-17.48 ± 93.28	-197.44
χ_4	CG-CD-CE-NZ	Lysine sidechain torsion 4	-77.44	-60.37 ± 7.24	17.06

Table S4: Comparison of retinal dihedral angles for an alternative **all-*trans*#2** isomer conformation: Molecular dynamics (MD) averages versus starting structure. Angles are reported in degrees (°).

**Dihedral Label**	**Atoms Involved**	**Description**	**Start. (°)**	**MD Avg. ± SD (°)**	**Δ (°)**
ϕ_1	C5-C6-C7-C8	6-7 bond torsion	152.10	132.25 ± 43.00	-19.84
ϕ_2	C6-C7-C8-C9	7-8 bond torsion	176.77	179.60 ± 9.94	2.83
ϕ_3	C7-C8-C9-C10	8-9 bond torsion	175.61	181.26 ± 8.49	5.64
ϕ_4	C8-C9-C10-C11	**9-10 bond**	221.91	183.37 ± 26.48	-38.53
ϕ_5	C9-C10-C11-C12	10-11 bond torsion	155.69	180.66 ± 15.98	24.97
ϕ_6	C10-C11-C12-C13	**11-12 bond**	198.92	183.30 ± 14.09	-15.62
ϕ_7	C11-C12-C13-C14	12-13 bond torsion	180.41	180.94 ± 7.27	0.53
ϕ_8	C12-C13-C14-C15	13-14 bond torsion	186.49	183.00 ± 12.00	-3.48
ϕ_9	C13-C14-C15-N	14-15-Schiff base torsion	176.20	159.50 ± 55.80	-16.70
χ_1	N-CA-CB-CG	Lysine sidechain torsion 1	-64.84	-57.89 ± 19.68	6.95
χ_2	CA-CB-CG-CD	Lysine sidechain torsion 2	171.89	178.64 ± 6.01	6.74
χ_3	CB-CG-CD-CE	Lysine sidechain torsion 3	-53.25	-40.66 ± 59.78	12.59
χ_4	CG-CD-CE-NZ	Lysine sidechain torsion 4	-58.00	-59.50 ± 6.49	-1.50

Table S5: Weights of each edge between pairs of residues along the communication paths.

Edge	9-*cis*	11-*cis*	all-*trans* #1	all-*trans* #2
Retinal → Trp290	2.83	1.60	3.07	0.91
Trp290 → Ala289	0.69	0.81	0.56	0.20
Ala289 → Met288	0.92	0.51	0.68	0.65
Met288 → Cys285	2.34	1.92	2.23	2.76
Cys285 → Cys284	0.38	0.45	0.38	0.66
Cys284 → Ile283	0.26	0.39	0.38	0.36
Ile283 → Thr282	0.49	0.45	0.40	0.50
Thr282 → Ala279	0.81	0.98	0.77	1.09
Ala279 → Ala276	1.42	1.46	1.34	1.67
Ala276 → Leu275	0.56	0.76	0.67	0.86
Leu275 → Glu272	3.31	3.11	2.46	2.82
Glu272 → Ala271	0.09	0.11	0.46	0.11
Ala271 → Ser270	0.18	0.11	0.14	0.09
Ser270 → Asn266	3.39	5.59	3.49	3.17
Asn266 → Asn263	1.47	0.71	0.60	0.68
Asn263 → Ser262	0.76	1.26	1.06	1.11
Ser262 → Ser259	1.30	1.25	1.28	1.34
Ser259 → Lys258	1.49	1.78	1.92	1.81
Path length (d_{min})	22.69	23.25	21.89	20.79
